# Phthalide Derivatives from *Angelica Sinensis* Decrease Hemoglobin Oxygen Affinity: A New Allosteric-Modulating Mechanism and Potential Use as 2,3-BPG Functional Substitutes

**DOI:** 10.1038/s41598-017-04554-3

**Published:** 2017-07-14

**Authors:** Wei-Ren Chen, Youqing Yu, Muhammad Zulfajri, Ping-Cheng Lin, Chia C. Wang

**Affiliations:** 1 Department of Chemistry, National Sun Yat-sen University, Kaohsiung, Taiwan 80424 Republic of China; 2 Aerosol Science Research Center, National Sun Yat-sen University, Kaohsiung, Taiwan 80424 Republic of China

## Abstract

*Angelica sinensis* (AS), one of the most versatile herbal medicines remains widely used due to its multi-faceted pharmacologic activities. Besides its traditional use as the blood-nourishing tonic, its anti-hypertensive, anti-cardiovascular, neuroprotective and anti-cancer effects have been reported. Albeit the significant therapeutic effects, how AS exerts such diverse efficacies from the molecular level remains elusive. Here we investigate the influences of AS and four representative phthalide derivatives from AS on the structure and function of hemoglobin (Hb). From the spectroscopy and oxygen equilibrium experiments, we show that AS and the chosen phthalides inhibited the oxygenated Hb from transforming into the high-affinity “relaxed” (R) state, decreasing Hb’s oxygen affinity. It reveals that phthalides cooperate with the endogenous Hb modulator, 2,3-bisphosphoglycerate (2,3-BPG) to synergetically regulate Hb allostery. From the docking modeling, phthalides appear to interact with Hb mainly through its α_1_/α_2_ interface, likely strengthening four (out of six) Hb “tense” (T) state stabilizing salt-bridges. A new allosteric-modulating mechanism is proposed to rationalize the capacity of phthalides to facilitate Hb oxygen transport, which may be inherently correlated with the therapeutic activities of AS. The potential of phthalides to serve as 2,3-BPG substitutes/supplements and their implications in the systemic biology and preventive medicine are discussed.

## Introduction

*Angelica sinensis* (AS), one of the most versatile and prevalent herbal medicines has been commonly used as a tonic to promote blood circulation, to alleviate migraines and gynecological disorders, and to treat mild anemia and hypertension^1–3^. Its anti-cardiovascular^3^, neuroprotective^4^, and anti-cancer^5–8^ effects have also been reported. Previous compositional analysis of AS have identified phthalide derivatives, ferulic acid and polysaccharides as the major constituents of AS, though their relative abundance may vary depending on the area and growth conditions^9–12^. Amongst, phthalide derivatives, featured by a γ-valerolactone attached to a six-membered carbon ring have been suggested as the bioactive components of AS and have been used as the marker to assess the quality of AS^9–12^. The therapeutic effects of several phthalide derivatives have been reported. For example, n-butylidenephthalide has been found to exhibit the anti-proliferative effect for tumor cells and has been suggested as a potential treatment for brain and colon cancers^5, 13^. Z-ligustilide, on the other hand, has been reported to play a neuroprotective role and has been suggested to treat neurodegenerative diseases, such as Alzheimer’s disease (AD)^14^. Though different constituents in AS may be involved in treating different medical conditions, however, the nearly panacea-like character of AS in remedying such diverse syndromes and diseases strongly hints that the bioactive components in AS may actively participate and intervene the systemic circulation, and a strong interplay exists between the bioactive components in AS and the key functional constituent in the blood.

**Figure 1:**
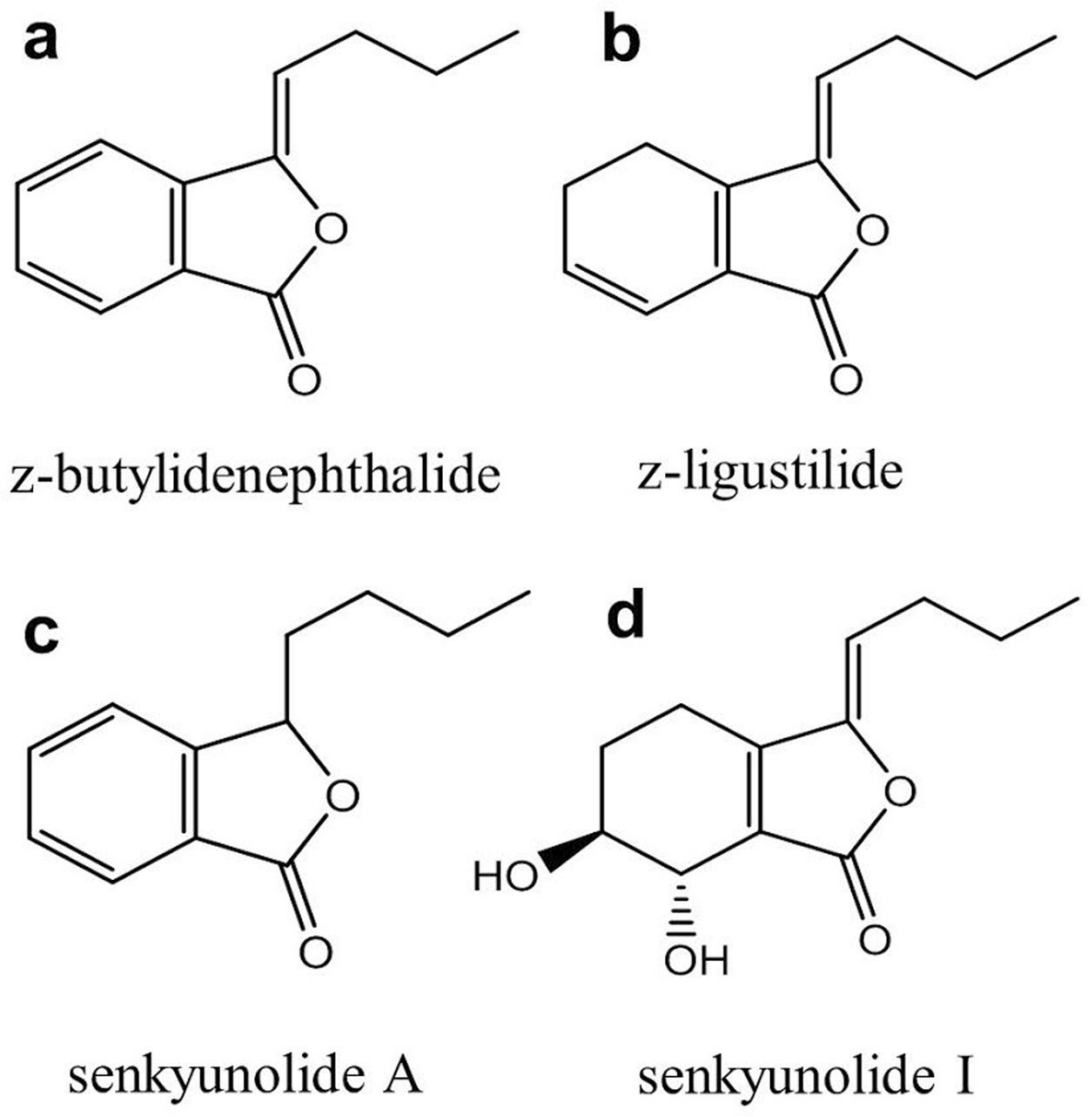
Molecular structure of four representative phthalide derivatives from AS. (**a**) z-butylidenephthalide (**b**) z-ligustilie (**c**) senkyunolide A (**d**) senkyunolide I.

To verify this hypothesis and gain insights into the molecular mechanism underlying the diverse efficacies of AS, here we investigate the influences of AS and four representative phthalide derivatives previously identified from AS, including z-butylidenephthalide (Fig. 1a), z-ligustilide (Fig. 1b), senkyunolide A (Fig. 1c), senkyunolide I (Fig. 1d) on the structure and oxygen transport function of hemoglobin (Hb).

Hb is the oxygen-carrying protein in erythrocytes responsible for transporting oxygen from lungs to tissue cells. It is a hetero-tetramer (α_2_β_2_) comprised of two α and two β subunits arranged tetrahedrally, with each subunit containing a heme group and a globular protein. The heme iron lies out of the porphyrin plane when deoxygenated, with the quaternary structure of deoxygenated Hb (deoxyHb) resided in the low oxygen affinity “tense” (T) state. Upon oxygenation, the binding of oxygen to the heme iron pulls the iron into the porphyrin plane, which causes certain spatial constraints to destabilize the T state and triggers the oxygenated hemoglobin (oxyHb) to transform into the high oxygen affinity, “relaxed” (R) state^15^. The Hb allosteric transition dynamics has been an area of extensive study^15–26^. Several models, including the MWC concerted model^15, 21^, the KNF sequential model^22^, Perutz’s stereochemical model^18, 20, 24^, and the global allostery model have been proposed to describe the mechanism of Hb allostery^23^. Time-resolved spectroscopic^27–30^ and crystallographic^26, 31^ studies have unraveled detailed allosteric transition dynamic of Hb in short time regimes ranging from sub-milliseconds to hundreds of femtoseconds. Recent time-resolved wide angle X-ray scattering experiments^31^ and conjugate peak refinement calculations^19^ further suggested that Hb allosteric transition is more complex than previously anticipated, involving multiple stages of quaternary changes. The influences of heterotropic effectors, such as H^+^, CO_2_ and the endogenous Hb modulator, 2,3-bisphosphoglycerate (2,3-BPG) in regulating Hb allostery and its oxygen affinity have been extensively studied^32–35^. The presence of 2,3-BPG is particularly essential to guide Hb to release oxygen properly, which is achieved by preferentially binding to the low affinity T state of Hb to decrease its oxygen affinity^36–38^. Though 2,3-BPG exists in erythrocytes with a relatively high *in vivo* concentration for normal adults (~5 to 8 mM), the 2,3-BPG metabolism may be degraded chronically^39^. Insufficient 2,3-BPG levels in turn may lead to defect Hb oxygen transport, cellular hypoxia and increased rates of various oxygen transport deficit induced-/related- diseases. Therefore, the significance of an effective Hb modulator is by no means less than Hb itself in transporting oxygen. For instance, a markedly reduced cerebral Hb oxygenation level was observed for patients with AD, causing improper activation of brain function in the degenerating brain area^40, 41^. Two recent studies further established the close correlations between age-related 2,3-BPG metabolism disorder and neurodegenerative diseases, including both AD and non-AD dementia^39, 42^.

Here we first investigate the influences of AS and four phthalide derivatives from AS on the allosteric structural properties of Hb via the resonance Raman (RR) spectroscopy. The liganded status of Hb upon phthalide treatment is clarified by the UV-visible absorption spectroscopy. We then evaluate the capacity of phthalide derivatives in modulating Hb’s oxygen affinity via the oxygen equilibrium experiments. The active sites of Hb upon interacting with phthalides are identified from the molecular docking modeling. A new Hb allosteric-modulating mechanism is proposed for the first time to interpret the superior allosteric-regulating capability of phthalides on Hb. Finally, the potentials of phthalide derivatives to be used as the 2,3-BPG functional substitutes/supplements to promote Hb oxygen transport and their possible implications in the system biology and preventive medicine are discussed.

## Results

### AS and phthalide derivatives inhibit oxyHb from converting into the high affinity R State

**Figure 2:**
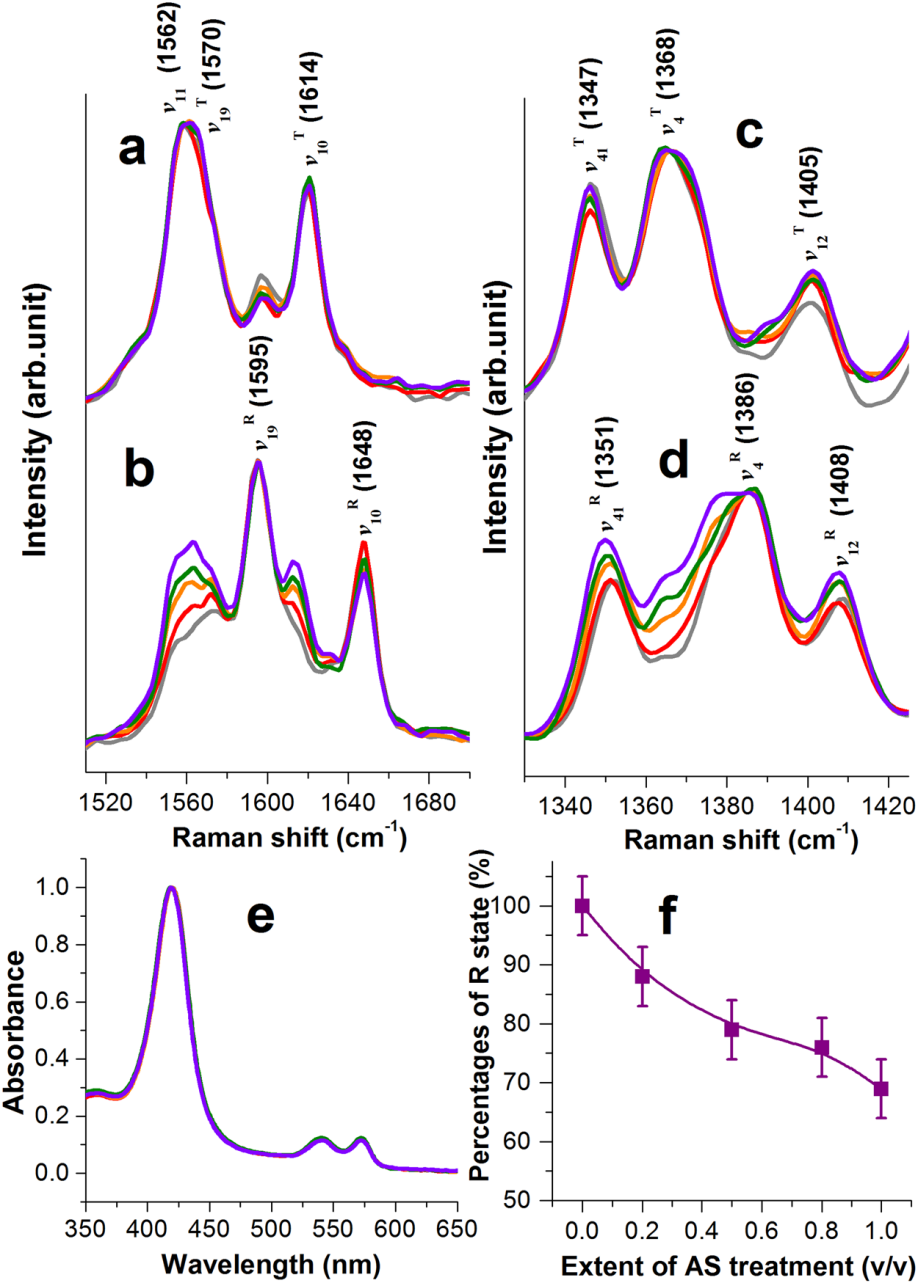
Influences of AS extract on Hb allosteric behaviors. RR spectra of pure and AS-treated Hb between 1500 and 1700 cm^−1^ measured under (**a**) a nitrogen atmosphere, and (**b**) an oxygen atmosphere. RR spectra of pure and AS-treated Hb between 1330 and 1425 cm^−1^ measured under (**c**) a nitrogen atmosphere, and (**d**) an oxygen atmosphere. (**e**) UV-visible absorption spectra of pure and AS-treated Hb under the oxygen atmosphere. Color code for (**a**) to (**e**): pure Hb (gray), AS-treated Hb with the “AS-to-Hb volume ratio” of 0.2 (red), 0.5 (orange), 0.8 (green), 1 (violet). (**f**) Percentages of R state of AS-treated Hb in the oxygen atmosphere at varying extents of AS treatment. The data was presented with the mean ± SEM between two independent measurements.

We first investigated the influence of AS plant extract on the structures of Hb via RR spectroscopy at 532 nm. Since the excitation wavelength is close in energy with the electronic transition of the heme group from its ground state to the first electronic excited state (S_0_ → S_1_), the Raman scattering signals are resonance-enhanced. The RR spectral features of purified Hb are dominated by the vibrations of heme group of Hb, which showed clear distinctions between the deoxyHb in the low affinity T state (Fig. 2a, gray curve) and oxyHb in the high affinity R state (Fig. 2b, gray curve)^43^. The most prominent bands for deoxyHb, measured under an atmosphere filled with nitrogen simulating a deoxygenated environment were the ν_11_ (B_1g_), ν_19_ (A_2g_) and ν_10_ (B_1g_) modes of the porphyrin ring centered at 1562, 1570 and 1614 cm^−1^, respectively (denoted ν_11_, ν_19_^T^ and ν_10_^T^)^44–46^. Replacing the atmosphere to that filled with oxygen, ν_19_ and ν_10_ shifted to 1595 cm^−1^ (denoted ν_19_^R^) and 1648 cm^−1^ (denoted ν_10_^R^) respectively. The ν_10_ and ν_19_ vibrations are associated with the C_α_-C_m_ and C_α_-C_β_ vibrations of the porphyrin ring. Upon oxygenation, the porphyrin ring of heme group transforms from the low affinity “out-of-plane” to the high affinity “in-plane” configuration, which reduced the core size of heme group and inevitably perturbed the local coordinates of the C_α_-C_m_ and C_α_-C_β_ of the porphyrin ring, leading to an increased force constant of these vibrations and the blue shift. On the other hand, the ν_11_ vibration, mostly originated from the C_β_-C_β_ stretching vibration did not exhibit observable shift but decreased in intensity upon oxygenation. A similar blue shift was also observed at the ν_4_ (A_1g_) band, the pyrrole half-ring symmetric vibration, which shifted from 1368 cm^−1^ for deoxyHb in the T state (gray curve in Fig. 2c, denoted ν_4_^T^) to 1386 cm^−1^ for oxyHb in the R state (gray curve in Fig. 2d, denoted ν_4_^R^). Due to the relative larger spectral shift between the T and R states, the ν_11_, ν_19_ and ν_10_ bands were used here to assess the configuration status of heme group and the corresponding structure of Hb.

We next investigated the RR spectra of Hb treated with varying extents of AS extract. For the RR spectra of AS-treated Hb under a deoxygenated environment (Fig. 2a), the relative intensity at 1595 cm^−1^ coinciding with ν_19_^R^ reduced as compared with pure Hb, suggesting that the porphyrin ring for AS-treated Hb converted more effectively into the “out-of-plane” configuration upon deoxygenation. On the other hand, the RR features of the AS-treated Hb under the oxygen atmosphere demonstrated an explicitly dose-dependent behavior (Fig. 2b). The relative intensities of ν_11_, ν_19_^T^ and ν_10_^T^ vibrations increased systematically with advancing degrees of AS-treatment, indicating that some phyto-chemical constituents in the AS plant extract has forced the heme group to retain in the low affinity “out-of-plane” configuration. Such a dose-dependent increase in the low affinity T state component was also revealed at the ν_4_ band for AS-treated Hb (Fig. 2d).

The increase of T state component for AS-treated Hb in the oxygen atmosphere may be originated from two possible scenarios. First, the heme iron for AS-treated Hb may have lost its ability to bind O_2_ upon the AS-treatment and Hb should reside in the “deoxy-T” state. Second, the heme iron may indeed be bound with O_2_, but the heme group remained in the low affinity, “out-of-plane” configuration and the protein retained in the T state. For the latter, the AS-treated oxyHb should reside in the “oxy-T” state. To clarify the liganded status of heme iron for AS-treated Hb under such an atmospheric condition, we measured the UV-visible absorption spectra of AS-treated Hb under the oxygen atmosphere (Fig. 2e). The doublet features centered at 541 and 573 nm, corresponding to the Q band (S_0_ → S_1_ transition) of Hb is characteristic for a liganded status of the heme iron^47^, verifying that the AS-treated Hb was indeed oxygenated under the oxygen atmosphere. All AS-treated Hb absorption spectra are plotted in superposition to reveal the consistent liganded status for all AS-treated Hb.

Without any external force, the heme groups tend to adapt the high affinity “in-plane” configuration upon oxygenation. Therefore, the increased proportion for AS-treated oxyHb to retain in the “out-of-plane” heme configuration indicated that certain external forces exerted by AS or its bioactive components have stabilized the oxygen-bound heme groups in the low affinity “out-of-plane” configuration. Since all six coordination sites of heme iron were already occupied, the only way to intervene heme conformation is through the allosteric regulation. This observation suggested that AS, or its bioactive constituents acted as potent Hb allosteric modulators, which interacted with Hb via its protein part to inhibit its transition into the high affinity R state, or stabilize oxyHb in the low affinity T state, thus maintaining the oxygen-bound heme group in the out-of-plane configuration. The R state inhibition effect of AS was evaluated in terms of the R state fraction for AS-treated oxyHb with increasing degrees of AS-treatment (Fig. 2f), achieved by fitting a simulated spectrum comprised of adjustable weighing factors of the T and R states of pure Hb to the observed RR spectrum of AS-treated Hb. The result showed that the R state proportion of AS-treated oxyHb decreased progressively with increasing degrees of AS-treatment. Because the ultimate mission of Hb is to deliver oxygen to tissues and organs, inability to release oxygen properly results into reduced cellular oxygenation and related malfunction of the body. The observed R state inhibition for AS-treated oxyHb indicated that oxygen became less tightly bound to Hb, indicating that oxygen may be released more efficiently to tissues and organs with the aid of AS.

**Figure 3:**
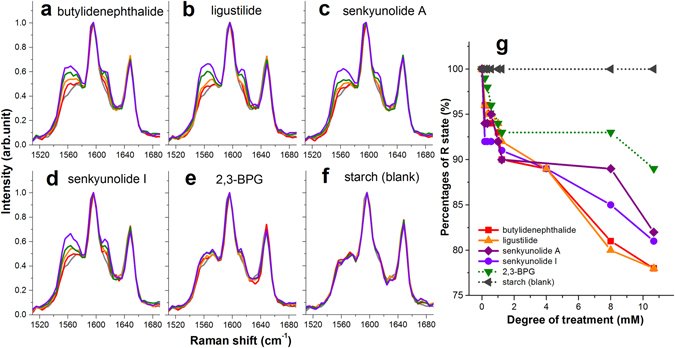
Influences of phthalide derivatives on Hb allosteric behaviors. RR spectra of Hb treated with (**a**) z-butylidenephthalide (**b**) z-ligustilide (**c**) senkyunolide A (**d**) senkyunolide I at varying levels of treatments under the oxygen atmosphere. RR spectra of Hb treated with (**e**) 2,3-BPG (endogenous Hb modulator) and (**f**) starch (blank experiment) were also measured as the references. Color code for (**a**) to (**f**): pure Hb (gray), Hb treated with the specified compound of: 1 mM (red), 4 mM (orange), 8 mM (green) and 12 (violet) mM. (**g**) Evolution of R state percentages for oxyHb treated with z-butylidenephthalide (red solid curve), z-ligustilide (orange solid curve), senkyunolide A (purple solid curve), senkyunolide I (violet solid curve), 2,3-BPG (green dash curve) and starch (gray dash curve) with increasing degrees of treatment.

While AS is a natural product containing numerous phyto-constituents^9^, previous compositional analysis suggested that phthalide derivatives, including ligustilides and senkyunolides are the bioactive species of AS^3, 5, 11, 13^. To clarify whether phthalide compounds are the bioactive species responsible for the observed R state inhibition effect on oxyHb, we independently assessed the effects of four representative phthalide derivatives of AS^9^, including z-butylidenephthalide, z-ligustilide, senkyunolide A and senkyunolide I on Hb, both in the oxygen atmosphere (Fig. 3a to d) and in the nitrogen atmosphere (Supplementary Fig. S1a to d).

The effect of 2,3-BPG on Hb was also characterized under the corresponding atmospheric conditions (Fig. 3e and Supplementary Fig. S1e) to serve as a reference, whereas the RR spectra of Hb treated with starch was acquired as the blank experiment (Fig. 3f and Supplementary Fig. S1f). With increasing degrees of phthalide treatments, progressively increasing proportions of phthalide-treated Hb were found to reside in the low affinity “out-of-plane” configuration in the oxygen atmosphere, confirming that the four representative phthalide derivatives of AS were capable of suppressing the high affinity R state of oxyHb. The liganded status of pure- and treated-Hb under the oxygen atmosphere was clarified also from both the UV-visible absorption spectra (Supplementary Fig. S2a to f) and near-infrared Band III absorption spectra (Supplementary Fig. S3a to f), confirming that the heme iron of phthalide-treated Hb was indeed bound with oxygen. The possibility that the observed RR spectral change is associated with the formation of methemoglobin (metHb) has been excluded upon evaluating the observed UV-visible spectra for all treated Hb which showed no evidence of presence of metHb^47^. Therefore, the observed R state inhibition for the phthalide-treated oxyHb can be attributed solely to the allosteric-modulatory effect of phthalide derivatives on Hb.

To quantitatively characterize the capacities of chosen phthalide derivatives in inhibiting the R state of oxyHb, the fractions of R state for oxyHb treated with the four phthalide derivatives at varying levels were extracted from the observed RR spectra (Fig. 3g), based on the same fitting procedure as that performed on AS-treated Hb. The observed oxy-R state suppression exerted by the chosen phthalides on oxyHb closely resembles that exerted by 2,3-BPG (green dash curve in Fig. 3g). On the other hand, for oxyHb treated with starch (blank experiment, gray dash curve in Fig. 3g), it converted completely to the R state, as expected. Such a dose-dependent R state reduction was also revealed consistently at the ν_4_ band region for phthalide-treated oxyHb under the oxygen atmosphere (Supplementary Fig. S4). This set of results showed explicitly that AS and the representative phthalides from AS exhibit pronounced allosteric-modulatory effects on Hb, and they modulate Hb allostery in a way similar to 2,3-BPG by stabilizing oxyHb in the low affinity T state. Nevertheless, some subtle but critical difference exists between phthalides and 2,3-BPG, as elaborated in Discussion.

### Phthalide derivatives facilitate Hb oxygen release by decreasing Hb oxygen affinity

**Figure 4:**
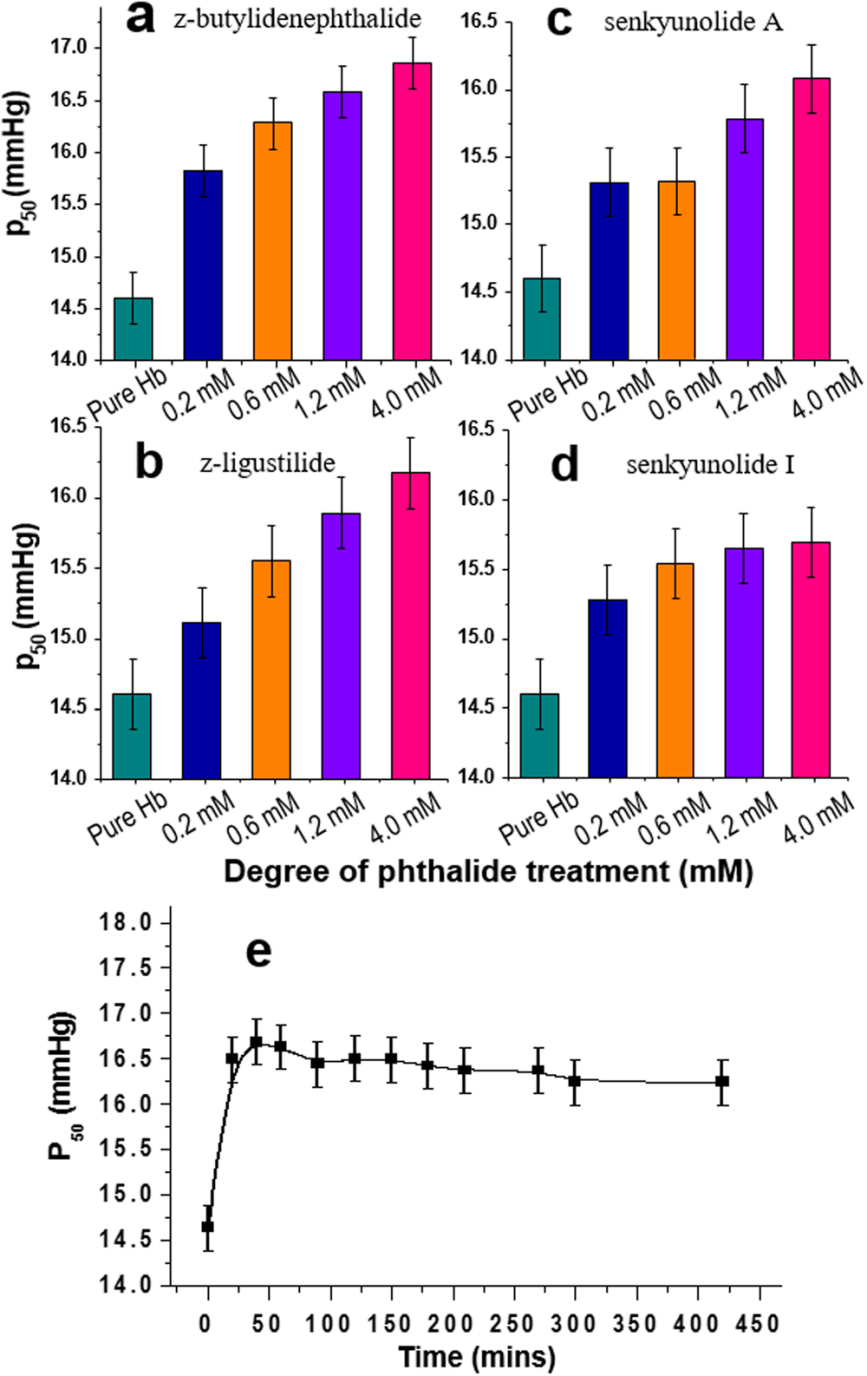
Effects of phthalide derivatives in decreasing Hb oxygen affinity. The P_50_ values for Hb treated with (**a**) z-butylidenephthalide (**b**) z-ligustilide (**c**) senkyunolide A (**d**) senkyunolide I at varying levels of treatments between 0.2–4.0 mM. The P_50_ for pure Hb was also shown as reference. (**e**) Time evolution of P_50_ for Hb treated with 4 mM of z-butylidenephthalide. The mean ± SEM of P_50_ was determined from four independent measurements of Hb.

The spectroscopic observation of phthalide derivatives to stabilize oxyHb in the low affinity T state indicated that the phthalide-treated Hb should carry oxygen with reduced oxygen affinity and release oxygen more readily. To verify this, we next performed the oxygen equilibrium curve (OEC) measurements to assess Hb oxygen affinity upon treating with varying levels of phthalide derivatives. By doing so, the P_50_ values for Hb treated with z-butylidenephthalide, z-ligustilide, senkyunolide A and senkyunolide I (Fig. 4a to d, respectively) at varying degrees of phthalide treatments, including 0.2 mM (Fig. 4, blue columns), 0.6 mM (Fig. 4, orange columns), 1.2 mM (Fig. 4, violet columns) and 4.0 mM (Fig. 4, pink columns) were extracted. The P_50_ value, defined as the oxygen partial pressure (pO_2_) required for Hb to become half oxygenated/deoxygenated is a direct measure of oxygen affinity for Hb. A higher P_50_ value indicates that a higher oxygen partial pressure is required for Hb to become half-saturated, thus corresponding to a lower oxygen affinity. The P_50_ value for Hb treated with corresponding levels of 2,3-BPG was also measured as a reference (Supplementary Fig. S5). In comparison with the P_50_ value for pure Hb at 14.60 ± 0.25 mmHg (Fig. 4, dark cyan columns), the P_50_ value for Hb treated with 1.2 mM of z-butylidenephthalide, z-ligustilide, senkyunolide A and senkyunolide I and 2,3-BPG was measured to be at 16.58, 15.89, 15.78, 15.65 and 18.05 ± 0.25 mmHg, respectively (Fig. 4, violet columns).

From the OEC analysis, all phthalide-treated Hb demonstrated a dose-dependent increase of P_50_ with advancing phthalide treatments (Fig. 4a to d). To assess the onset time for the phthalide to begin taking effect, the temporal evolution of the P_50_ value for the phthalide-treated Hb was also measured (Fig. 4e, using 4 mM of z-butylidenephthalide for example), which showed that z-butylidenephthalide began to exert the Hb modulating effect nearly immediately after it was added to Hb (within 30 min, the detection time limit for the OEC measurements). This set of results clearly show that these representative phthalide derivatives from AS can lower the Hb oxygen affinity and facilitate the oxygen release from Hb in a timely and efficient manner.

### Phthalide derivatives cooperate with 2,3-BPG synergetically

**Figure 5:**
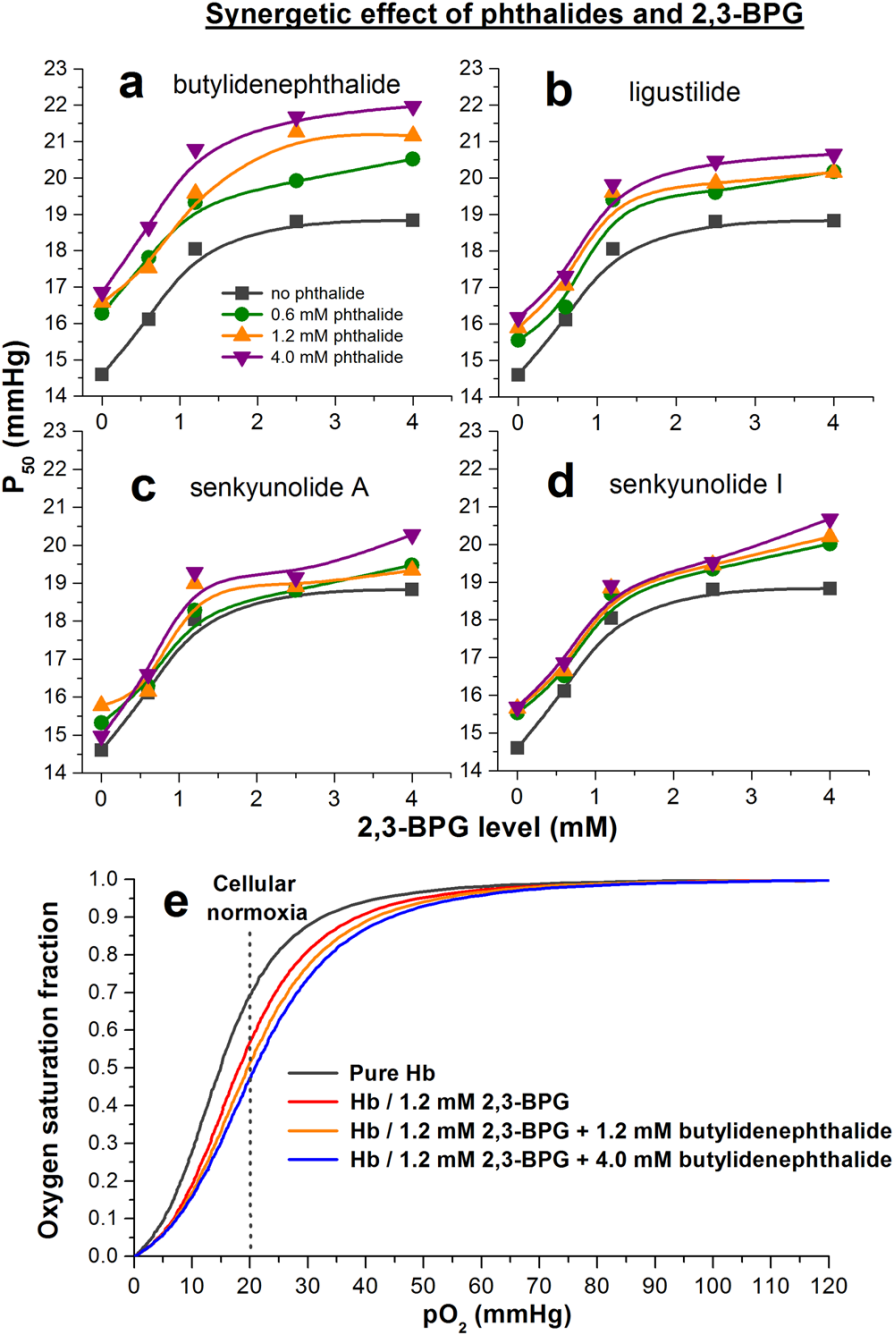
Synergetic effects of phthalide derivatives and 2,3-BPG in modulating Hb allostery. The P_50_ values for Hb treated with (**a**) z-butylidenephthalide, (**b**) z-ligustilide, (**c**) senkyunolide A, and (**d**) senkyunolide I of 0.6 mM (green curves), 1.2 mM (orange curves) and 4.0 mM (purple curves) at varying 2,3-BPG levels. The P_50_ evolution for Hb treated with 2,3-BPG only (gray curves) is also shown as reference. (**e**) OEC for: pure Hb (gray curve), Hb treated with 1.2 mM 2,3-BPG (red curve), Hb treated with 1.2 mM 2,3-BPG and 1.2 mM z-butylidenephthalide (orange curve), and Hb treated with 1.2 mM 2,3-BPG and 4.0 mM z-butylidenephthalide (purple curve).

While 2,3-BPG plays an essential role in assisting Hb to release oxygen properly^36–38^, the 2,3-BPG metabolism may degrade with aging^39^. Once the *in vivo* 2,3-BPG level falls below the normal physiological range (~5 to 8 mM), the insufficient 2,3-BPG levels may lead to impaired Hb oxygen transport, increased cellular hypoxia, and increased incidence rates of various oxygen transport deficit related diseases. On the other hand, even if the 2,3-BPG level is reduced due to chronic degradation, it never completely depletes in erythrocytes. Therefore, it is important to assess whether the phthalide derivatives retain their Hb modulating abilities under the presence of 2,3-BPG, or even remedy the impaired Hb oxygen transport function when the 2,3-BPG level is too low to meet the physiological requirement (<5 mM). We performed the OEC measurements and obtained the P_50_ values for Hb treated with z-butylidenephthalide (Fig. 5a), z-ligustilide (Fig. 5b), senkyunolide A (Fig. 5c) and senkyunolide I (Fig. 5d) under the presence of varying levels of 2,3-BPG. Three levels of phthalide treatments were applied to evaluate their abilities to cooperate with 2,3-BPG in modulating the P_50_ values for the treated Hb, including 0.6 mM (Fig. 5, green curves), 1.2 mM (Fig. 5, orange curves) and 4.0 mM (Fig. 5, purple curves) of the specified phthalides, which were then directly compared with Hb treated only with 2,3-BPG (Fig. 5, gray curves). The results clearly showed that the four phthalide derivatives can indeed cooperate with 2,3-BPG to raise the P_50_ values for Hb under the presence of 2,3-BPG.

It is particularly profound to note that the phthalide-treated Hb can reach a similar P_50_ value at reduced 2,3-BPG levels. For instance, without treating with any phthalides, it necessitates a 2,3-BPG level of 4 mM for Hb to reach a P_50_ value of 18.83 ± 0.25 mmHg. However, when Hb was treated with 0.6~4.0 mM of phthalides, it can reach a similar or even higher P_50_ level at considerably lower 2,3-BPG levels, implying that the phthalide derivatives are capable of serving as 2,3-BPG substitutes/supplements when needed. The permeability of phthalide derivatives to RBC were also investigated by tracking the P_50_ values of RBC treated with two phthalides (Supplementary Fig. S6), which showed that phthalides can permeate through RBC to modulate the oxygen affinity of Hb.

Due to the distinct metabolic requirements, different organs and tissue cells exhibit different physiological pO_2_ requirements^48^. The extent of oxygen release enhancement of phthalides for different organs and tissues may be accessed by tracking the oxygen saturation fraction along the OECs at the corresponding physiological pO_2_ values (Fig. 5e). For example, at pO_2_ of 20 mmHg which corresponds to the cellular normoxia, the fraction of oxygen-bound Hb under 1.2 mM 2,3-BPG is 0.57 (Fig. 5e, dark gray curve), indicating that 43% of Hb can release oxygen and reside in the unbound deoxyHb form. However, with presence of 4.0 mM z-butylidenephthalide (Fig. 5e, purple curve), the fraction of oxygen-bound Hb at pO_2_ of 20 mmHg dropped to 0.48, indicating that the percentages of Hb allowed to release oxygen increased to 52%, corresponding to an enhancement factor of 1.21. It is also crucial to note that while the phthalide derivatives efficiently enhance the Hb oxygen release, they do so without sacrificing Hb’s oxygen uptake capacities in the lung, as manifested from the oxygen saturation fraction of phthalide-treated Hb which remained highly oxygen saturated (>0.95) at pO_2_ relevant to alveolus of ~100–120 mmHg. This observation suggests that when the 2,3-BPG levels of individuals become insufficient to meet the physiological oxygen demands, or additional oxygen is required to alleviate the cellular hypoxia conditions, phthalide derivatives are highly promising to serve as 2,3-BPG functional substitutes/supplements to modulate the OEC in such a way that the oxygen demands of different organs and tissues can still be met at reduced 2,3-BPG levels. The P_50_ values and the Hill’s coefficient, n_50_ for all phthalide- and 2,3-BPG-treated Hb are summarized in Supplementary Table S1, where the n_50_ value is a measure of Hb cooperativity. From the relatively stable n_50_ values for the phthalide-treated Hb, the cooperativity of Hb is maintained upon the treatment of phthalides.

### Phthalides interact with Hb via its α_1_/α_2_ interface

To learn how the T state of oxyHb can be stabilized upon the phthalide treatment, we docked^49^ these phthalide derivatives individually to the crystalline structure of Hb in the T state, first by keeping the protein rigid while allowing the bond angles and torsion angles of phthalide molecule to be flexible. Only the low affinity T state of Hb was chosen for the molecular docking modeling here because this is the only relevant structure to access how the phthalide molecule come into play to inhibit Hb from converting to the R state. Two Hb T state coordinates were used, including that reported by Fermi *et al*. (PDB 2HHB, 1.74 Å resolution)^50^ and the more recent one by Park *et al*. (PDB 2DN2, 1.25 Å resolution)^51^. Despite some minor difference, the molecular docking results from both Hb T state consistently revealed α_1_Arg141 as the main active site of Hb, along with some other residues near the α_1_/α_2_ interface (Table 1, the 1^st^ and 2^nd^ columns). The docking modeling was also performed for 2,3-BPG to assure the rigidity of the docking protocol since the binding sites of 2,3-BPG to Hb T state have been reported from the crystallographic study (at least to 3.5 Å resolution)^52^. The computationally identified active sites of Hb upon docking with 2,3-BPG are found to situate at the entrance of the β_1_/β_2_ cavity, in satisfactory agreement with that reported crystallographically by Arnone^52^. In specific, the previously suggested 2,3-BPG binding sites of βVal1, βLys82 and βHis143 are consistently identified from the present docking modeling (Table 1). For the docking modeling of the higher resolution coordinates, 2DN2 (1.25 Å), βAsn139 is registered as the binding site instead of βHis143; however, this does not change the fact that 2,3-BPG plugs into the central cavity between the two β subunits of Hb. The coordinates with the PDB codes of 2HHB and 2DN2 are specifically chosen to represent the low affinity Hb T structure of two varying resolutions. Because the primary goal to perform the docking modeling here is to clarify how the phthalides intervene to inhibit the Hb transition into the R state, coordinates of oxygen-bound Hb that are already resided in the R state or Hb in any other liganded forms, such as Hb in the carbonmonoxy (Hb-CO) or nitric monoxide (Hb-NO) forms are not relevant and cannot provide the necessary information to clarify how the low affinity T state can be possibly stabilized upon treating with phthalides.

**Table 1:**
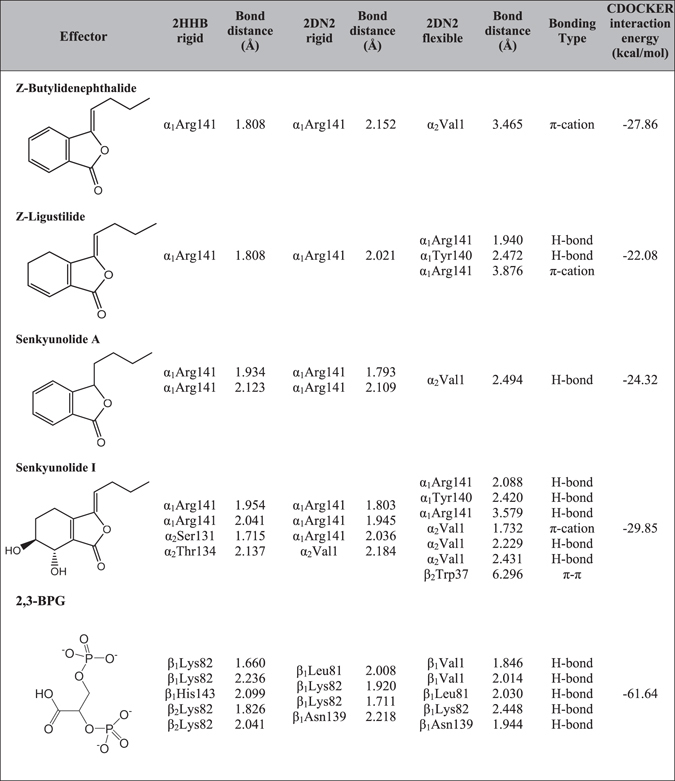
Summary of the molecular docking modeling results for the four chosen phthalide derivatives and 2,3-BPG docked to the available Hb T states.

**Figure 6:**
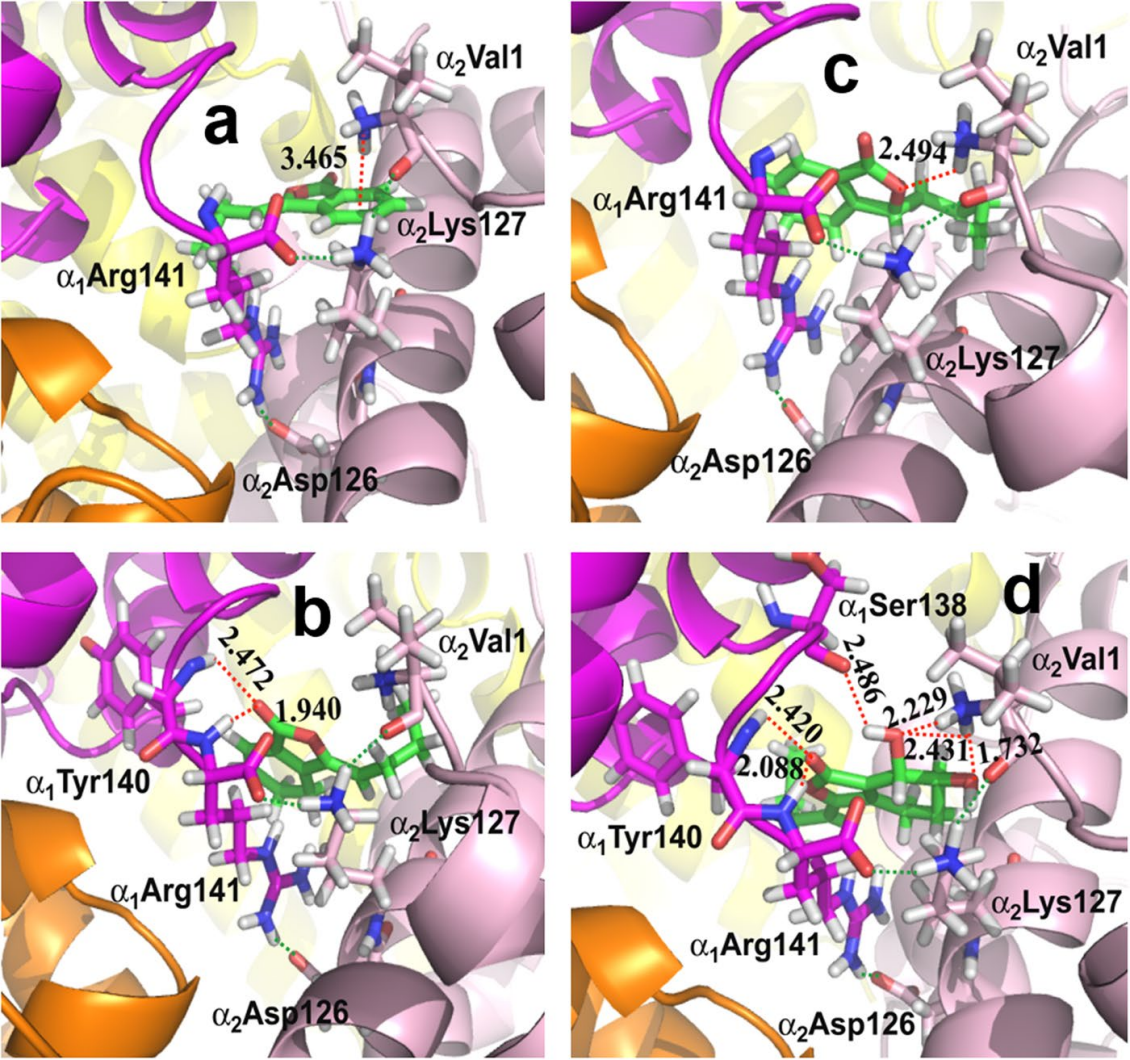
Intermolecular interactions between Hb and the phthalide compounds. (**a**) z-butylidenephthalide (**b**) z-ligustilide (**c**) senkyunolide A (**d**) senkyunolide I identified from the flexible docking modeling. The new hydrogen bonds formed between Hb and the phthalide molecule are expressed as red dashed lines, while the hydrogen bonds that already exist are shown as green dashed lines. The bond distance is specified in Å. Color code: the backbone of all phthalides (green), α_1_ subunit and the backbone of its residues (magenta), α_2_ subunit and the backbone of its residues (light magenta), β_1_ subunit (light yellow), β_2_ subunit (orange), oxygen (red), nitrogen (blue) and hydrogen (white). Note that all four phthalide derivative compounds appear to interact with Hb between α_1_ (magenta) and α_2_ (light magenta) subunits.

To further investigate the effect of side chain motions of the active residues on the binding sites, we then performed the flexible docking modeling on the higher resolution Hb T state, allowing the side chains of the above-identified active residues to be flexible. The flexible docking modeling (Table 1, the 3^rd^ column) consistently revealed the α_1_/α_2_ interface as the active region, and α_1_Arg141 and α_2_Val1 as two important residues of Hb upon Hb-phthalide interactions. The flexible docking models for z-butylidenephthalide (Fig. 6a), z-ligustilide (Fig. 6b), senkyunolide A (Fig. 6c) and senkyunolide I (Fig. 6d) were illustrated, revealing their local intermolecular interaction network. To further validate the identified active sites upon the Hb-phthalide interaction, we further performed the docking modeling for six additional phthalide derivatives that were also identified previously in AS^9^, including 3-butylphthalide, e-ligustilide, senkyunolide F, senkyunolide H, 3-butylidene-4-hydrophthalide and 6,7-dihydroxyligustilide (Supplementary Table S2 and Supplementary Fig. S7), which consistently revealed α_1_Arg141 and α_2_Val1 as the two most crucial active sites. Altogether with the above four phthalide derivatives, the molecular docking modeling results for the total 10 phthalide derivatives confirmed that the Hb α_1_/α_2_ interface is the active region, and α_1_Arg141 and α_2_Val1 are the two critical residues involved in the Hb-phthalide interaction.

## Discussions

**Figure 7:**
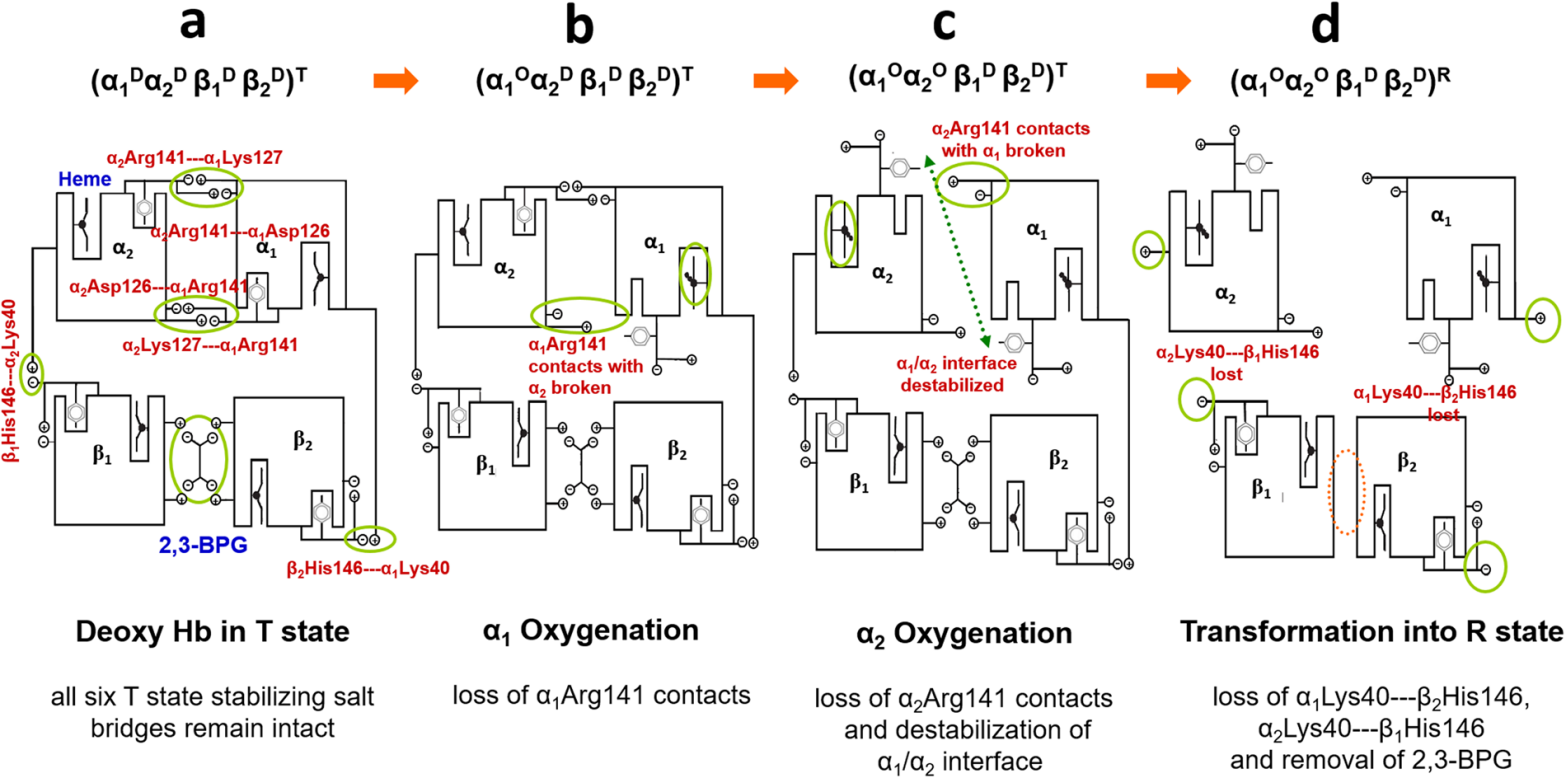
Illustration of six T state stabilizing salt bridges in Hb T-R transition. The Hb T-R transition at the early oxygenation stages and the status of six T state stabilizing salt bridges, as proposed by Perutz are shown. All six T state stabilizing salt bridges, including α_1_Arg141–α_2_Asp126, α_1_Arg141–α_2_Lys127, β_1_His146–α_2_Lys40 and their symmetrical counterparts, α_1_Asp126–α_2_Arg141, α_1_Lys127–α_2_Arg141 and β_2_His146–α_1_Lys40 are highlighted to illustrate the status of each T state stabilizing salt bridge during progressive oxygenation. (**a**) DeoxyHb in the T state with all six T state stabilizing salt bridges remained intact, (**b**) oxygenation occurs on α_1_ subunit, with the α_1_Arg141–α_2_Asp126, α_1_Arg141–α_2_Lys127 salt bridges ruptured, (**c**) oxygenation occurs on α_2_ subunit, with α_2_Arg141–α_1_Asp126, α_2_Arg141–α_1_Lys127 salt bridges ruptured. Hb’s α_1_/α_2_ interface becomes destabilized. (**d**) The last two T state stabilizing salt bridges are ruptured and 2,3-BPG is repelled out of the β_1_/β_2_ cavity. The role of αArg141 in constituting the α_1_/α_2_ interface and in sustaining the low-affinity T state can be visualized.

**Figure 8:**
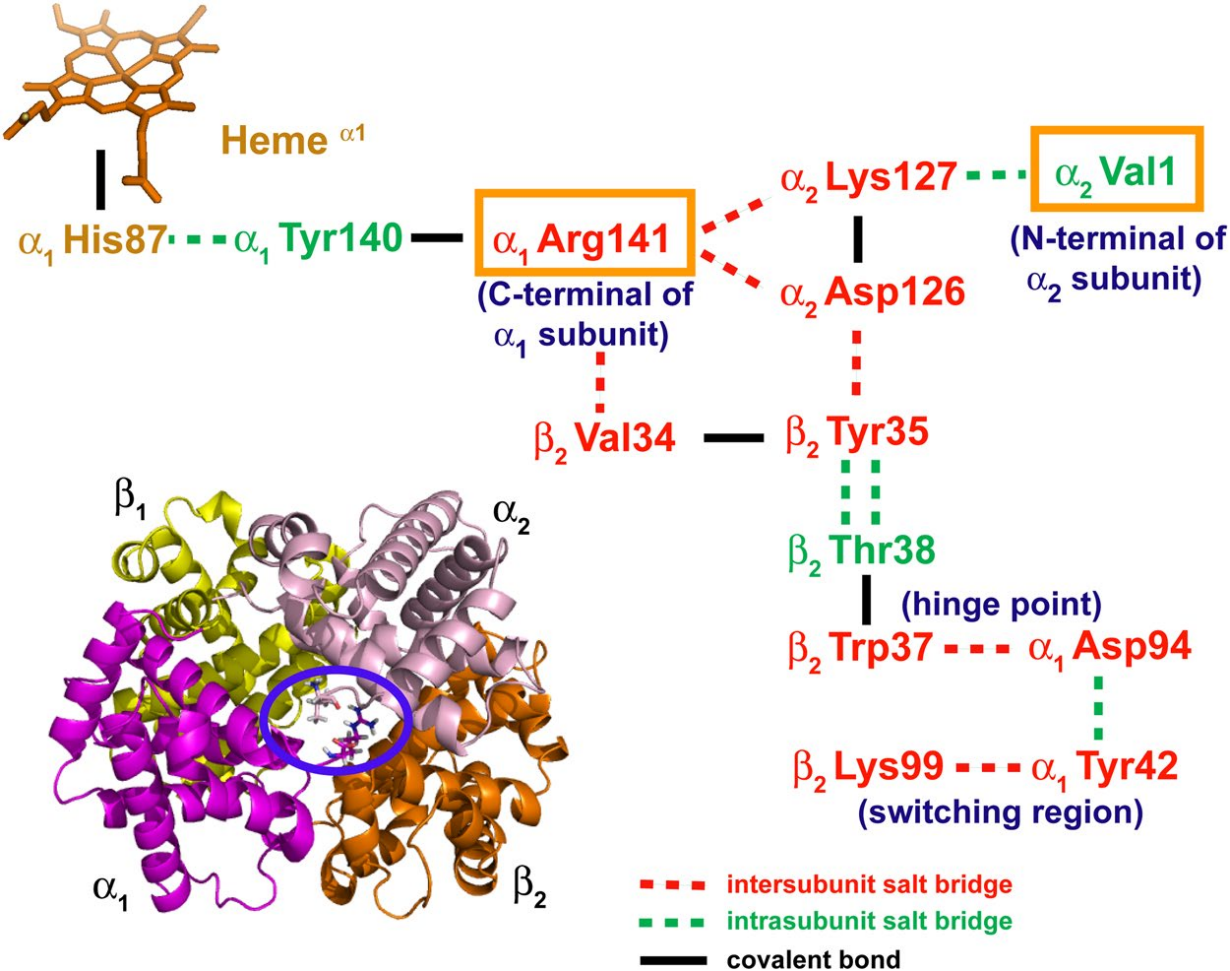
Illustration of the significance of α_1_Arg141 and α_2_Val1 in Hb allostery. The close correlations of α_1_Arg141, α_1_Tyr140, α_1_His87 and other crucial residues in the Hb allosteric transition are illustrated. The active area between the two α subunits is highlighted with a blue circle in the Hb crystal structure at the left bottom. All “inter-subunit” salt bridges are designated as red dash lines, while all “intra-subunit” salt bridges are expressed as green dash lines. The covalent bonds are specified as black solid lines. Color code: residues participated in the inter-subunit salt bridges: red; residues involved in the intra-subunit salt bridges: green; the proximal histidine and α-heme group: orange. Note the close association between α_1_Arg141 and the proximal histidine, α_1_His87 (and therefore the α-heme group), as well as the essential role of α_1_Arg141 in constituting the α_1_/α_2_ interface by forming the T state stabilizing α_1_Arg141–α_2_Asp126 and α_1_Arg141–α_2_Lys127 inter-subunit salt bridges (and their symmetric counterparts, α_2_Arg141–α_1_Asp126 and α_2_Arg141–α_1_Lys127). α_2_Val1 is also closely correlated with the α_1_/α_2_ interface through the α_2_Val1–α_2_Lys127 intra-subunit salt bridge. The close associations of αArg141 with two previously identified allosterically-crucial salt bridges, α_1_Asp94–β_2_Trp37 (the hinge point) and α_1_Tyr42–β_2_Lys99 (the switching region) are also revealed.

By examining the locations of the crucial residues identified from docking modeling with respect to the entire Hb tetramer, their extraordinary significance unveils. αArg141, the C-terminus of α globin is the most crucial residue in constituting Hb’s α_1_/α_2_ interface. It forms the α_1_/α_2_ interface with the opposite α subunit via the inter-subunit salt bridges, α_1_Arg141–α_2_Asp126 and α_1_Arg141–α_2_Lys127 and their symmetrical counterparts, α_1_Asp126–α_2_Arg141 and α_1_Lys127–α_2_Arg141 (Fig. 7a). These four α_1_/α_2_ inter-subunit salt bridges account for four out of the six “T state stabilizing” salt bridges proposed by Perutz^18^. Since αArg141 is also right next to the penultimate tyrosine αTyr140, which forms an intra-subunit salt bridge with the proximal histidine αHis87 (Fig. 8), it is also highly sensitive to the oxygenation status of α subunit.

It has been well established that the Hb oxygenation begins from α subunits^18, 53, 54^. According to Perutz’s stereochemical model^18^, once both α-heme groups become oxygenated, the two αTyr140 residues get expelled out of the tyrosine pocket, which consequently rupture the four T state-constraining salt bridges associated with αArg141 and thus the Hb α_1_/α_2_ interface (Fig. 7b and c)^18^. Due to the close correlation between α_1_Tyr140 and α_1_Arg141, α_1_Tyr140 is inevitably affected by the Hb-phthalide interaction through its neighboring α_1_Arg141. Therefore, it is likely through the α_1_Tyr140–αHis87 salt bridge the heme configuration and thus the oxygen affinity of heme iron are modulated by phthalide compounds. Figure 8 illustrates the close correlations of α_1_Arg141, α_1_Tyr140, αHis87 and other crucial residues in the Hb allosteric transition.

The destabilization of Hb α_1_/α_2_ interface eventually leads to the rupture of the last two T-constraining salt bridges, β_1_His146–α_2_Lys40 and β_2_His146–α_1_Lys40, resulting into the shrinkage of the β_1_/β_2_ cavity and removal of 2,3-BPG from the β_1_/β_2_ cavity (Fig. 7d). It is only till this point oxyHb clicks from the quaternary T state to the quaternary R state, which is then followed by subsequent oxygenation of β subunits^18^. Both crystallographic^55^ and spectroscopic^56^ studies reported previously that deletion of αArg141 removes the quaternary constraints and increases Hb oxygen affinity, pointing to the unusual functional significance of αArg141. As for α_2_Val 1 (N-terminus of α globin), even though it is not directly involved in constituting the α_1_/α_2_ interface, it forms an intra-subunit H-bond with α_2_Lys127, the other constituting residue of the crucial α_1_Arg141–α_2_Lys127 salt bridge (Fig. 8).

It is noted that Perutz and colleagues investigated the binding of bezafibrate (BZF) and LR16 to deoxyHb crystallographically and concluded that these molecules bind to the sites between α subunits^57, 58^. Two other BZF derivatives, RSR-4 and L35 were also suggested to bind with Hb via αArg141^59, 60^. Taking the present findings along with these earlier studies into consideration, it strongly indicates that Hb’s α_1_/α_2_ interface is an allosterically-sensitive site highly vulnerable to be intervened by heterotropic modulators. Considering the remarkable significance of αArg141 in sustaining the T state, we anticipate that the T state stabilizing effect exerted by the phthalide derivatives is most likely achieved by forming additional intermolecular interaction(s) with αArg141 to reinforce the quaternary constraints to stabilize oxyHb in the T state. Since additional energy is required to break the additional constraints contributed by the Hb-phthalide interaction, the Hb α_1_/α_2_ interface becomes more difficult to be ruptured and the transition of Hb into the R state becomes less readily to occur. Similarly, by forming intermolecular interactions with α_2_Val1, it also puts additional constraints to strengthen the α_1_/α_2_ interface, likely through the H-bond it shared with α_2_Lys127. As a result, by forming additional intermolecular interactions at these critical sites, Hb’s α_1_/α_2_ interface becomes fortified and the transition of oxyHb to the R state is inhibited.

Even though both 2,3-BPG and phthalides lower the Hb oxygen affinity by stabilizing the T state, it is instructive to compare the mechanisms from which they interact with Hb. From the previous crystallographic study^52^ and the present docking modeling, 2,3-BPG docks into Hb’s β_1_/β_2_ cavity. In contrast, phthalide derivatives interact with Hb via its α_1_/α_2_ interface. The 2,3-BPG binding sites situates in the close vicinity of βHis146, another critical residue in Hb allostery^18, 61, 62^. The β_1_His146–α_2_Lys40 and β_2_His146–α_1_Lys40 inter-subunit salt bridges account for the last pair of T state stabilizing salt bridges (Fig. 7a)^18^. According to Perutz’s model^18^, β_1_His146–α_2_Lys40 is substantial in keeping the penultimate β_1_Tyr145 fixed in the tyrosine pocket. Therefore, by plugging into the β_1_/β_2_ cavity, 2,3-BPG likely stabilizes the T state by strengthening the last two T-constraining salt bridges, β_1_His146–α_2_Lys40 and β_2_His146–α_1_Lys40. However, 2,3-BPG eventually gets repelled out of the β_1_/β_2_ cavity as soon as the α_1_/α_2_ interface becomes destabilized (Fig. 7c and d), and oxyHb eventually converts into the high affinity R state^18^. In contrast, phthalides appear to stabilize oxyHb in the T state by strengthening the α_1_/α_2_ interface via forming additional intermolecular interactions with αArg141. The relative significance of αArg141 and βHis146 in sustaining the T state has been studied by comparing the stability of Hb T state with either αArg141^55^ or βHis146^63^ removed. It was reported that αArg141 have a stronger impact in affecting the stability of T state than βHis146. Considering that Hb oxygenation begins from α subunits, strengthening the α_1_/α_2_ interface appears to be an efficient route to stabilize the T state, in contrast to the conventional pathway taken by 2,3-BPG via the β_1_/β_2_ cavity.

Besides the traditional usage as the blood-nourishing tonic, AS has also been reportedly used for treating migraines, gynecological disorders, mild anemia and hypertension^2, 3^. Its neuroprotective^4^ and anti-cancer^5–8^ effects have also been documented. It is of profound significance to note that these above-mentioned syndromes/diseases are commonly linked with the malfunction of Hb oxygen transport and thus cellular hypoxia to varying extents (see Supplementary Information). Even though AS is one of the most versatile and prevalently used herbal medicines since ancient times, how AS can exert such diverse therapeutic activities remain unclear from the molecular level. From the present investigation, we anticipate that the multi-faceted therapeutic effects of AS are likely associated with a universal molecular origin – the capability of AS and specifically, its bioactive phthalide derivatives to allosterically modulate Hb to decrease Hb’s oxygen affinity. By doing so, Hb’s oxygen transport function can be significantly enhanced.

Illnesses often occur when the homeostasis of the body becomes out of balance, resulting into dysfunction of certain parts of the body. The concepts of systems biology and preventive medicine have emerged recently^64^. Instead of using the isolated biomarkers to diagnose and treat diseases at specific areas, the systems biology considers the entire body as a biological system and emphasizes greatly the inter-connectivity between different components in the body. From the present study, it reveals that the various seemingly uncorrelated efficacies of AS are very likely associated with a universal root cause- the Hb oxygen transport promoting effect exerted by the bioactive phthalide derivatives in AS via their potent Hb allosteric regulatory effect. Upon administration of AS, organs and tissue cells can be supplied with sufficient oxygen to execute their biological functions. In turn, the rates for the body dysfunction, and developments of diseases can be reduced. This readily reveals the significance of AS and its bioactive phthalides in promoting the homeostasis of the body as a whole biological system and in preventing syndromes and diseases related with defect oxygen transport from occurring.

In summary, this work reports the capabilities of phthalide derivatives from AS in promoting the oxygen transport function of Hb via allosterically stabilizing oxyHb in the low affinity T state. The active sites of Hb upon interacting with phthalides are identified via the molecular docking modeling, from which a new Hb allosteric-modulating mechanism is proposed, for the first time, to rationalize the capacity of AS and its bioactive phthalides to decrease Hb oxygen affinity and facilitate Hb oxygen transport. It has been an ongoing effort to seek for effective Hb modulators to regulate the Hb oxygen affinity, with the goals to improve the cellular oxygenation, to treat acute hypoxia and to prolong the shelf life of stored erythrocytes. In light of their pronounced Hb allosteric regulatory effects, the bioactive phthalide derivatives from AS appear promising to serve as 2,3-BPG functional substitutes/supplements to assist Hb in better releasing oxygen when the endogenous 2,3-BPG levels becomes too low to meet the physiological demands. The new Hb allosteric-modulating mechanism proposed here sheds new lights for one to develop new strategies to treat and/or prevent various Hb oxygen transport defect related syndromes and diseases. Further investigations are warranted to evaluate the bioavailability, *in vivo* efficacy and cytotoxicity of phthalides for the findings to be applied ultimately to human.

## Methods

### Materials

Starch, sodium phosphate monobasic (anhydrous, ≧99%) and sodium phosphate dibasic (anhydrous, ≧99%) were purchased from Sigma-Aldrich (St. Louis, MO, USA). 2,3-Bisphosphoglycerate (2,3-BPG) in the form of pentasodium salt was purchased from Santa Cruz Biotechnology (Dallas, TX, USA). Dried roots of *Angelica Sinensis* (Oliv.) Diels were purchased from Yong-Fong Herbal Medicines Inc. (Kaohsiung, Taiwan), while the four phthalide derivatives experimentally investigated in this work, including Z-butylidenephthalide (HPLC grade, >98%), z-ligustilide (HPLC grade, >98%), senkyunolide A (HPLC grade, >98%), senkyunolide I (HPLC grade, >98%) were purchased from Shanghai Yuanye Bio-Technology (Shanghai, China). The AS plant extract was prepared from the dried AS root by immersing 1 g of the dried AS root into 10 mL of DI water after initial cleaning and incubated for 20 minutes at 90 °C to obtain the concentrated AS plant extract of 2.5 mL. This plant extract preparation method was chosen as this is the most common way from which the roots of AS was prepared as the traditional tonic^1, 65, 66^. The resulting plant extract was separated from the plant material and further centrifuged at 12,000 × g for 10 minutes to remove any residual fine particulates.

### Preparation of Pure and Treated Hemoglobin

The blood samples donated from healthy volunteers were collected in accordance with the guidelines approved by the Institutional Review Board of Kaohsiung Medical University Chung-Ho Memorial Hospital (IRB project number: KMUH-IRB-20120280), with the participants signed informed consent. Hb was purified from the drawn human blood^67^. In brief, the erythrocytes after separated from plasma were washed four times with 0.9% NaCl solution. The packed erythrocytes were mixed with one volume of water and dialyzed overnight at 4°C. Purified Hb solution was obtained by centrifuging the dialyzed hemoglobin solution at 18000 × g for 20 minutes to remove any particulate matters. The concentration of Hb was 3.8 × 10^−4^ M (pH = 7.4, 1X PBS buffer), as determined spectrophotometrically at 576 nm with an extinction coefficient of 10.07 L/mmol·cm^47^. An aliquot of 100 μl of the purified Hb solution of 3.8 × 10^−4^ M was then treated with the above prepared AS plant extract with varying AS-to-Hb volume ratios (v/v). The treatments of various compounds on Hb, including z-butylidenephthalide, z-ligustilide, senkyunolide A, senkyunolide I, starch and 2,3-BPG were performed by mixing varying volumes of the specified effector of 2.4 × 10^−2^ M with 100 μl of the purified Hb solution of 3.8 × 10^−4^ M. The final effector concentrations after mixing with Hb range between 0.2 and 12 mM. For the investigations on the permeability and modulating capacities of phthalide compounds to RBC (Figure S5), the phthalide stock solutions of 20, 70 and 120 μl were administered to 40 μl of RBC (denoted as “Low”, “Medium” and “High” levels of phthalide treatment in Figure S5, respectively). All treated Hb samples were incubated at 4°C for at least 12 hours before the spectroscopic and OEC measurements.

### Resonance Raman spectroscopy

The RR spectroscopy of pure and treated Hb was performed at 532 nm excitation (alpha 300, WITec Inc., Germany). For all RR spectroscopic measurements, 50 μL aliquot of each sample was placed onto a piece of SiO_2_ glass and a custom-made glass cover shield was used to enclose the detection area with continuously purged nitrogen or oxygen gas of ultrahigh purity. The power of the excitation laser (Diode-pumped Excelsior CW laser, Spectra Physics, CA, USA) was kept at sub mW to avoid photo-damages or thermal-degradation on the samples. The spectral features for each sample were continuously monitored to ensure that the structure of the sample was stabilized under the specified condition. The spectral resolution of all RR spectra was 4 cm^−1^. The acquisition time for each sample was kept at 10 s to assure the integrity of the sample. The RR spectra illustrating the ν_11_, ν_19_ and ν_10_ band region were normalized over the spectral range between 1480 and 1750 cm^−1^, while the RR spectra illustrating the ν_41_, ν_4_ and ν_12_ band region were normalized between 1300 and 1480 cm^−1^ to better reveal the spectral evolution with varying experimental conditions.

### UV-visible absorption spectroscopy

The UV-visible absorption spectra of pure and treated Hb were measured with the purpose to clarify the liganded status of the heme group under the specified conditions. A broadband light source (Energetiq EQ99-FC, Ocean Optics Inc., USA) and a TEC-cooled spectrometer (QE65Pro, Ocean Optics, Inc., USA) equipped with a FFT-CCD image sensor (S7031-1006, Hamamatsu) were used to acquire the UV-visible absorption spectra with a spectral resolution of 0.8 nm. For each UV-visible absorption measurement, 30 μL of each sample was diluted with 3 mL of DI water and measured in a quartz precision cell with an optical path of 10 mm (Hellma GmbH &Co., Germany). The acquisition time for each spectrum was kept at 100 ms. Oxygen gas of ultrahigh purity was introduced into the quartz cell and constantly purged for at least 10 minutes before the spectroscopic characterization.

### Oxygen equilibrium experiments

The oxygen equilibrium curves of purified- and treated- Hb samples were measured using the Hemox Analyzer (TCS Scientific Corporation, PA, USA) at 37 °C. For each measurement, 500 μL aliquot of Hb sample was mixed with 4 mL of 1X PBS buffer solution (pH = 7.4) and 10 μL of anti-foaming agent. Prior to the measurements, the Hb samples were allowed to saturate to the oxygen partial pressure, pO_2_ of 149 ± 1 mmHg by continuously purging with the compressed air. A Clark oxygen electrode was used to measure pO_2_ in the sample cuvette. Once Hb was fully saturated with oxygen, the purging gas was switched to pure N_2_ gas to deoxygenate the Hb sample and the oxygen saturation fraction was measured spectrophotometrically by recording the absorbance of oxy- and deoxy-Hb as a function of pO_2_.

### Molecular Docking Modeling

The 10 phthalide derivative compounds and 2,3-BPG were generated at the DFT//B3LYP/6-31 + G(d) level using Gaussian 03 W and docked to the available crystalline structures of Hb in the T state (PDB code: 2HHB^50^ and 2DN2^51^) using the AutoDock program, utilizing the Lamarckian Genetic Algorithm (LGA) and a scoring function which takes into accounts the van der Waals interactions, hydrogen bonding, torsion terms, electrostatic interactions to determine the ligand binding site and energy^49^. The native three-dimensional structure of Hb at the T state (PDB: 2HHB and 2DN2) was first set rigid, while the docking compounds were allowed to adjust the bond angles and torsion angles. The grid size was set to 126, 126, and 126 along X-, Y-, and Z-axis with 0.375 Å grid spacing. The parameters used for the docking models: GA population size = 150; maximum number of energy evaluations = 250,000; GA crossover mode = two points. One hundred docking runs were carried out in each case, and the resulting docked structures were clustered with a 2 Å tolerance for the rmsd. The validity and reliability of this docking protocol was verified by comparing the predicted binding sites of 2,3-BPG to Hb with the previous crystallographic study^52^. To access the effect of side chain motions on the binding sites of ligands to proteins, the flexible docking modeling was also performed using the CDOCKER, a grid-based MD docking algorithm^68^. The constituting residues of the α_1_/α_2_ interface and those resided in its close vicinity were chosen as the active side chain residues, including αArg141, αVal1, αLys99, αSer102, αAsp126, αAsp127, αSer131, αSer133, αThr134, βVal34. The energy minimization for each ligand molecule was computed using the conjugate gradient method with the CHARMm force field. The obtained poses were analyzed, and the highest score structure was chosen for the analysis of the interaction between ligand and Hb. The PyMOL software was used for visualization of the docked conformations.

## Electronic supplementary material

Supplementary Information
