# Supplementary material for: Phthalide Derivatives from Angelica Sinensis Decrease Hemoglobin Oxygen Affinity: A New Allosteric-Modulating Mechanism and Potential Use as 2,3-BPG Functional Substitutes
Source: Sci Rep. 2017 Jul 14;7:5504. doi: 10.1038/s41598-017-04554-3 (PMC5511246; doi:10.1038/s41598-017-04554-3)
Supplement: Supplementary file 1 — Supplementary Information [file 41598_2017_4554_MOESM1_ESM.pdf]

Phthalide Derivatives from *Angelica Sinensis* Decrease Hemoglobin Oxygen Affinity: A New Allosteric-Modulating Mechanism and Potential Use as 2,3-BPG Functional Substitutes

Wei-Ren Chen, Youqing Yu, Muhammad Zulfajri, Ping-Cheng Lin & Chia C. Wang*

Supplementary Information:

Correlations between Hb oxygen transport deficiency and some common diseases

Though most illnesses and diseases show different symptoms and seem irrelevant, many of them are commonly originated from or associated with the same root cause- the Hb oxygen transport defect. For instance, migraine is a collection of neurological symptoms originated from a change of blood flow in the brain. Once being triggered, certain spasms are created to restrict the nerve-rich scalp and carotid arteries supplying blood to the brain, reducing the amount of oxygen delivered to the brain.[1,2] Dysmenorrhea, a common menstrual disorder experienced by more than half of women population worldwide occurs because the prostaglandins produced by the endometrium cells during the menstruation period cause intensified uterine contraction which restricts the blood flow and reduces the oxygen supply to the uterus, causing the menstrual cramps.[3,4] As for hypertension, while it may be caused by genetic, environmental or even unknown reasons, patients suffering from hypertension commonly experience increased resistance of peripheral blood flow. To fulfill the oxygen demands and assure the biological functions can be performed correctly, the heart must work harder to increase the peripheral blood flow rate, which inevitably elevates the blood pressure. As first proposed by Marlow, a more intrinsic cause of hypertension should be attributed to defect oxygen transport.[5] The correlations between the cerebral hypoxia and neurodegenerative diseases have also been established. Hock *et al*[6] and Arai *et al*[7] have monitored the Hb oxygenation level in the frontal and parietal cortex for patients with Alzheimer's disease (AD) during the verbal fluency tasks. It was found that patients with AD experienced markedly reduced regional cerebral blood flow and decreased cerebral oxygenation level, causing improper activation of brain function in the degenerating brain area. Two recent studies established the close correlation between age-related 2,3-BPG

metabolism disorder and neurodegenerative diseases, including both AD and non-AD dementia.[8,9] The strong linkage between the low cellular oxygenation level and development of cancers has been long established. Despite the numerous secondary causes, the single primary cause of cancers is the low cellular oxygenation levels, as pointed out by Warburg.[10] A previous low-field paramagnetic imaging study also revealed unambiguously the significantly lower cellular oxygenation of tumor bearing mice than the normal ones.[11]

Supplementary Figures:

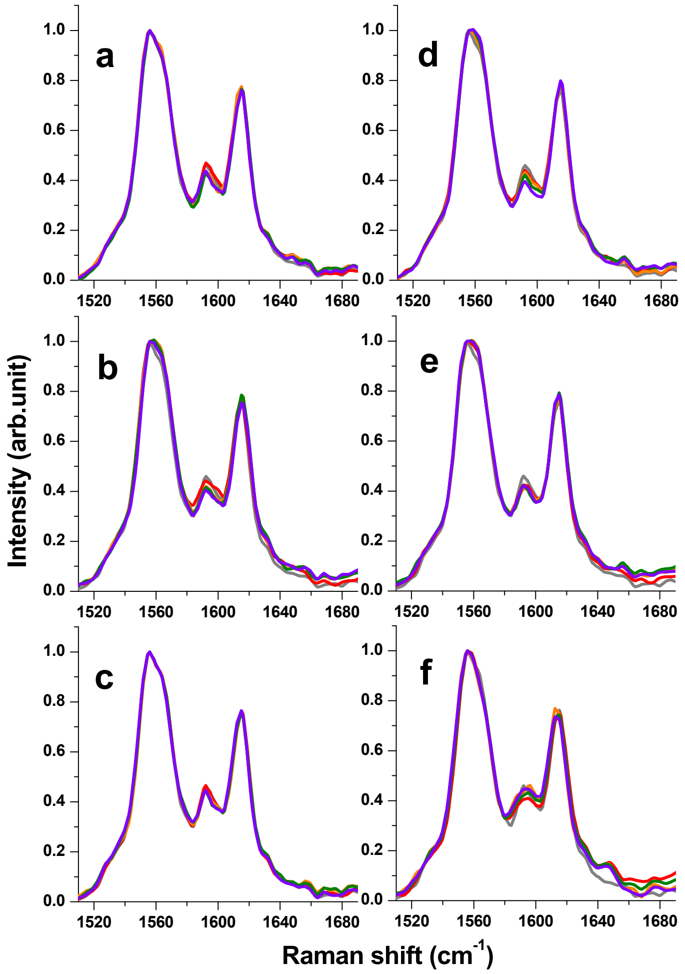

Supplementary Figure S1. RR spectra of Hb treated with varying levels of phthalides under the nitrogen atmosphere. (**a**) z-butylidenephthalide (**b**) z-ligustilide (**c**) senkyunolide A (**d**) senkyunolide I (**e**) 2,3-BPG (**f**) starch. Color code: pure Hb (gray), Hb treated with the specified compound of: 1 mM (red), 4 mM (orange), 8 mM (green) and 12 (violet) mM.

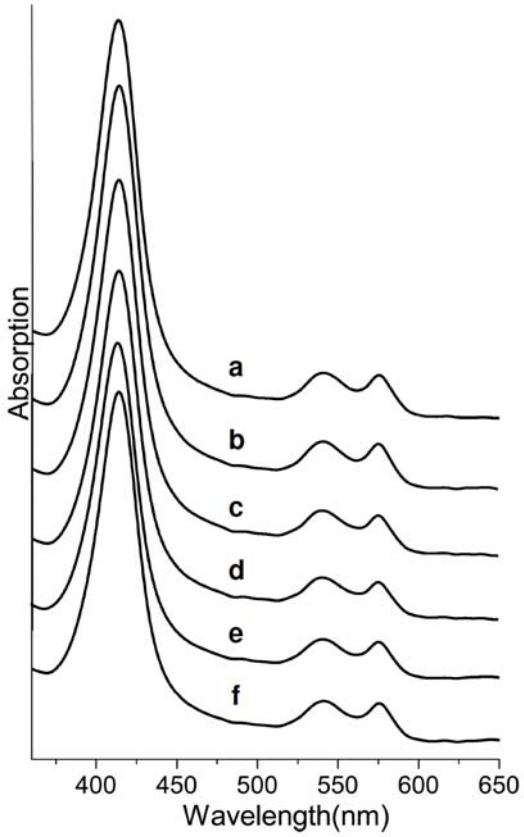

Supplementary Figure S2. UV-visible absorption spectra verify the liganded status for treated-Hb under the oxygen atmosphere. UV-visible spectra of Hb treated with 8 mM of (**a**) z-butylidenephthalide (**b**) z-ligustilide (**c**) senkyunolide A (**d**) senkyunolide I (**e**) 2,3-BPG (endogenous Hb modulator) and (**f**) starch (blank experiment) under the oxygen atmosphere. All treated Hb showed the doublet features at the Q band ($S_0 \rightarrow S_1$) at 541 and 573 nm, confirming that the treated Hb was indeed bound with oxygen under the oxygen atmosphere.

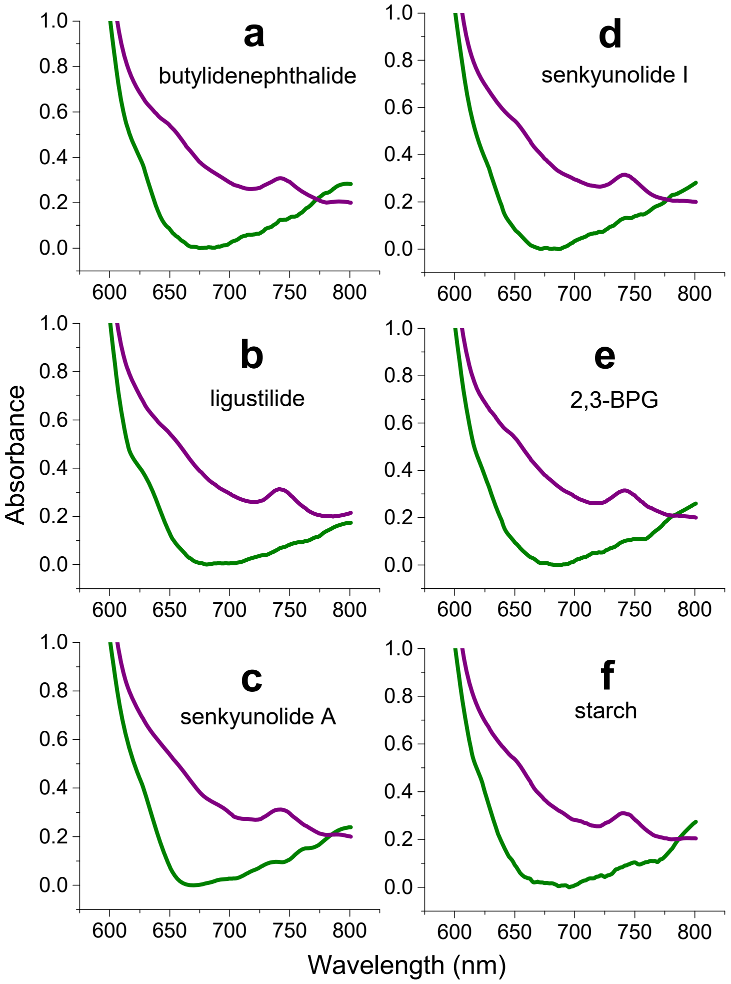

Supplementary Figure S3. Near-IR absorption spectra verify the liganded status for treated-Hb under the oxygen atmosphere. Near-infrared absorption spectra of Hb treated with 8 mM of (**a**) z-butylidenephthalide (**b**) z-ligustilide (**c**) senkyunolide A (**d**) senkyunolide I (**e**) 2,3-BPG (endogenous Hb modulator) and (**f**) starch (blank experiment) under the oxygen atmosphere (green curve) and nitrogen atmosphere (orange curve). Since Band III absorption at ~ 750 nm only appears for the five-coordinated ferrous hemes and is absent for the six-coordinated liganded hemes,[12] the absence of Band III absorption serves as an alternative indication for liganded heme groups. All treated Hb showed the absence of Band III under the oxygen atmosphere, a characteristic feature for the six-coordinated hemes, confirming that the treated Hb was bound with O_2 under the oxygen atmosphere.

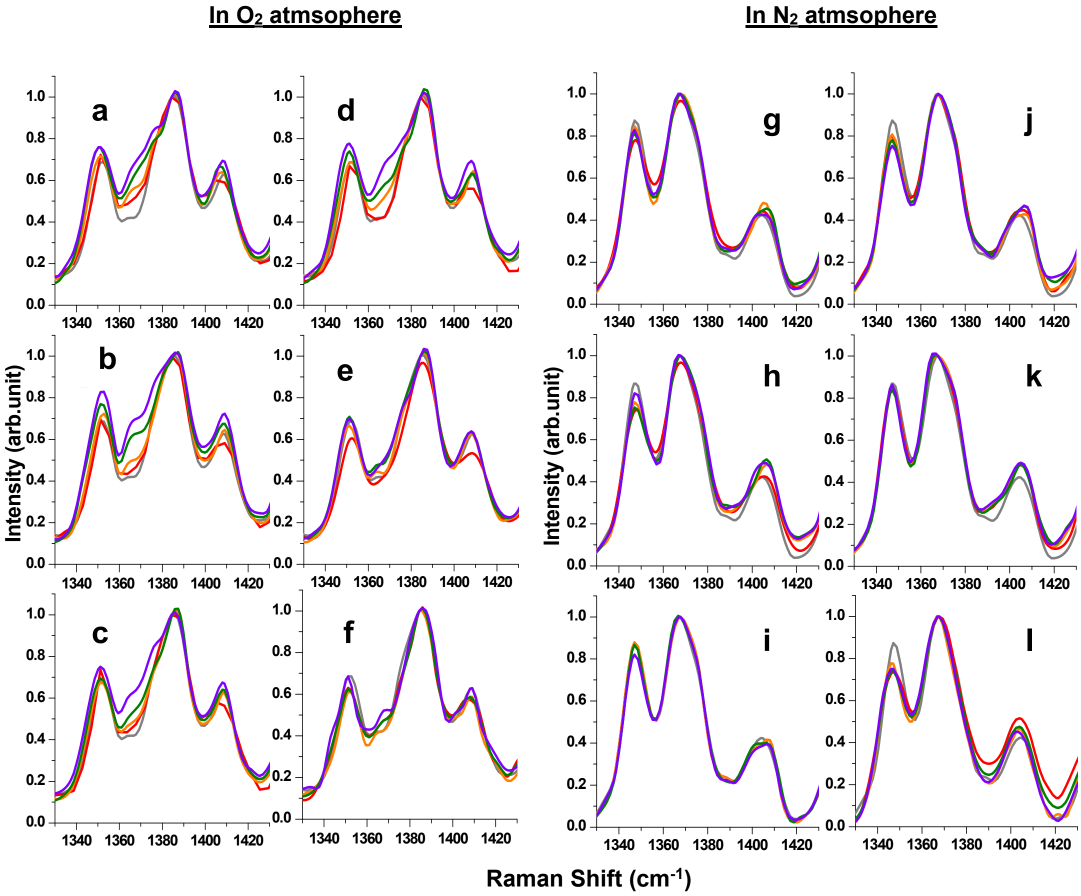

Supplementary Figure S4. RR spectra of treated Hb in the ν4 band region. The RR spectra of Hb treated with varying degrees of (**a**) z-butylidenephthalide (**b**) z-ligustilide (**c**) senkyunolide A (**d**) senkyunolide I (**e**) 2,3-BPG (**f**) starch measured under the oxygen atmosphere illustrating the ν4 band region. The RR spectra of Hb treated with (**g**) z-butylidenephthalide (**h**) z-ligustilide (**i**) senkyunolide A (**j**) senkyunolide I (**k**) 2,3-BPG (**l**) starch at varying levels of treatments under the nitrogen atmosphere were also shown. Color code: pure Hb (gray), Hb treated with the specified compound of: 1 mM (red), 4 mM (orange), 8 mM (green) and 12 mM (violet).

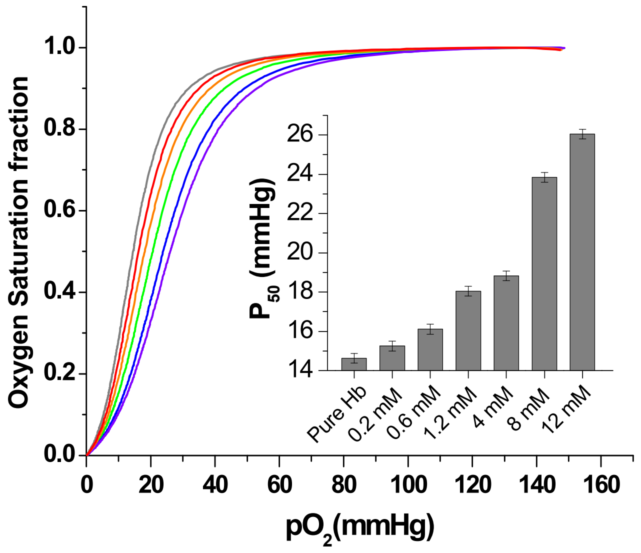

Supplementary Figure S5. The evolution of oxygen equilibrium curves for Hb treated with 2,3-BPG at varying levels. Color code: 0.6 mM (red curve), 1.2 mM (orange curve), 4 mM (green curve), 8 mM (blue curve) and 12 mM (violet curve). The OEC of pure Hb (gray curve) was also shown as the reference. The inset shows the evolution of P_{50} values for Hb treated with varying levels of 2,3-BPG extracted from the obtained OECs.

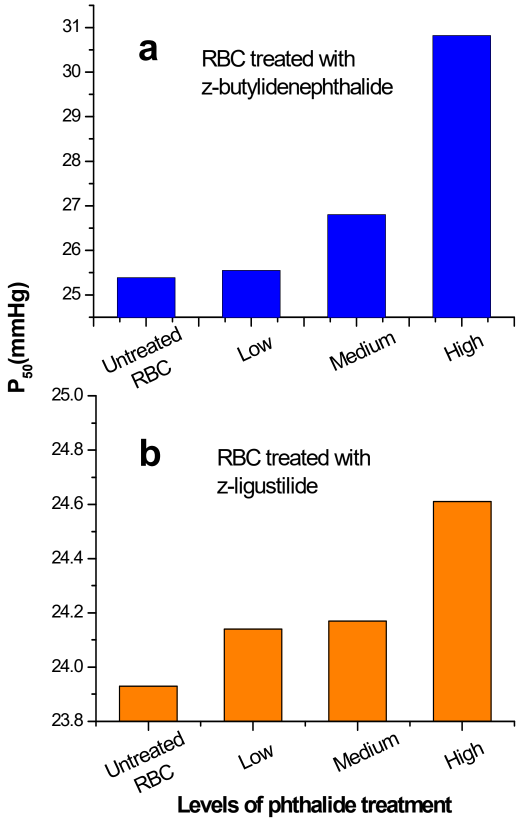

Supplementary Figure S6. The P_{50} values for RBC treated with phthalides at three varying levels of treatments. The P_{50} values for RBC treated with three different levels of (**a**) z-butylidenephthalide (**b**) z-ligustilide. Note that since there are relatively high levels of 2,3-BPG inherently existed in RBCs, the P_{50} values for RBC are higher than the purified Hb. Also, because the 2,3-BPG level in RBC degrades progressively over time, the P_{50} values for RBC treated with (**a**) z-butylidenephthalide and (**b**) z-ligustilide measured at different dates are for the purpose solely to demonstrate their permeability to RBC and cannot be directly compared with each other.

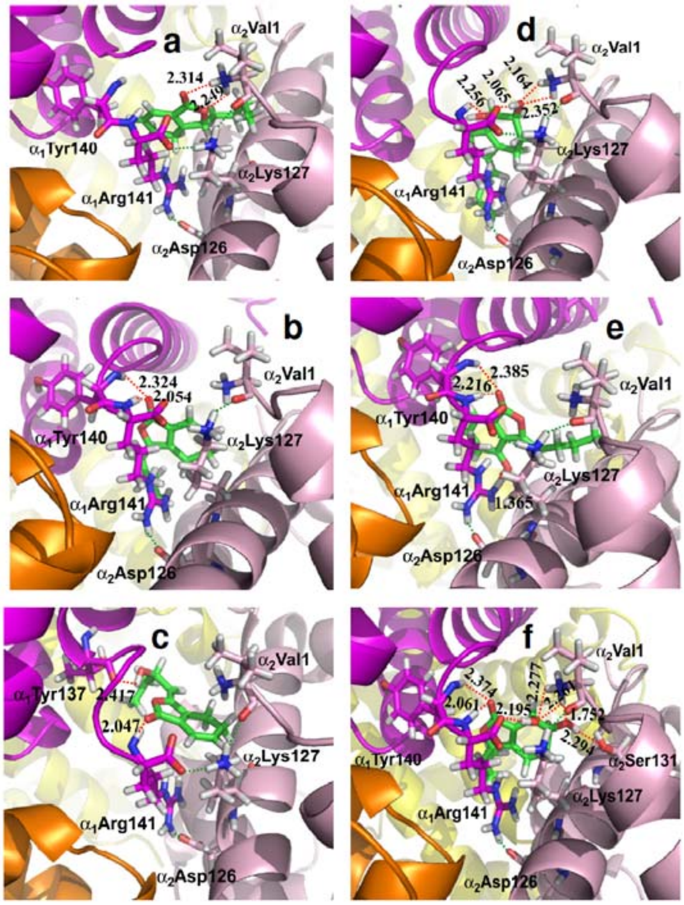

Supplementary Figure S7. **The flexible docking models illustrate the interactions between Hb and six additional phthalide derivatives.** (**a**) 3-butylphthalide (**b**) E-ligustilide (**c**) senkyunolide F (**d**) senkyunolide H (**e**) 3-butylidene-4-hydrophthalide (**f**) 6,7-dihydroxyligustilide. α_1 and α_2 denote the two α subunits when both α subunits were involved in forming the hydrogen bonds. The new hydrogen bonds formed between the phthalide compound and Hb are shown as red dashed lines, while the hydrogen bonds that already exist are shown as green dashed lines. The bond distance is specified in the unit of Å. Color code: the backbone of all phthalides (green); α_1 subunit and the backbone of its residues (magenta); α_2 subunit and the backbone of its residues (light magenta); β_1 subunit (yellow); β_2 subunit (orange); oxygen (red); nitrogen (blue) and hydrogen (white).

Supplementary Tables:

Supplementary Table S1. P_{50} and n_{50} values for phthalide-/2,3-BPG- treated Hb at varying conditions. (P_{50} unit in mmHg)

	no phthalide		0.6 mM phthalide		1.2 mM phthalide		4.0 mM phthalide	
2,3-BPG level	P_{50}	n_{50}	P_{50}	n_{50}	P_{50}	n_{50}	P_{50}	n_{50}
**Z-Butylidenephthalide**								
no 2,3-BPG	14.60	2.69	16.28	2.74	16.58	2.69	16.86	2.63
0.6 mM 2,3-BPG	16.11	2.73	16.75	2.70	17.53	2.64	18.65	2.62
1.2 mM 2,3-BPG	18.05	2.75	19.32	2.72	19.58	2.75	20.79	2.73
2.5 mM 2,3-BPG	18.80	2.67	19.92	2.82	21.27	2.77	21.68	2.74
4.0 mM 2,3-BPG	18.83	2.74	20.52	2.76	21.16	2.78	21.97	2.72
**Z-Ligustilide**								
no 2,3-BPG	14.60	2.69	15.55	2.71	15.89	2.68	16.17	2.67
0.6 mM 2,3-BPG	16.11	2.73	16.46	2.77	17.06	2.72	17.31	2.61
1.2 mM 2,3-BPG	18.05	2.75	19.40	2.74	19.60	2.70	19.82	2.73
2.5 mM 2,3-BPG	18.80	2.67	19.60	2.80	19.86	2.77	20.46	2.76
4.0 mM 2,3-BPG	18.83	2.74	20.17	2.77	20.15	2.74	20.65	2.69
**Senkyunolide A**								
no 2,3-BPG	14.60	2.69	15.32	2.69	15.78	2.71	14.98	2.72
0.6 mM 2,3-BPG	16.11	2.73	16.30	2.85	16.15	2.71	16.60	2.70
1.2 mM 2,3-BPG	18.05	2.75	18.28	2.67	18.99	2.72	19.29	2.70
2.5 mM 2,3-BPG	18.80	2.67	18.81	2.77	18.91	2.72	19.15	2.85
4.0 mM 2,3-BPG	18.83	2.74	19.48	2.33	19.34	2.74	20.28	2.74
**Senkyunolide I**								
no 2,3-BPG	14.60	2.69	15.54	2.79	15.65	2.75	15.69	2.70
0.6 mM 2,3-BPG	16.11	2.73	16.51	2.75	16.65	2.74	16.87	2.76
1.2 mM 2,3-BPG	18.05	2.75	18.69	2.73	18.84	2.73	18.92	2.76
2.5 mM 2,3-BPG	18.80	2.67	19.35	2.70	19.46	2.80	19.53	2.74
4.0 mM 2,3-BPG	18.83	2.74	20.02	2.81	20.21	2.81	20.68	2.80

Supplementary Table S2. Summary of the molecular docking modeling results for six additional phthalide derivatives docked to the available Hb T states.

Effector	2HHB rigid	Bond distance (Å)	2DN2 rigid	Bond distance (Å)	2DN2 flexible	Bond distance (Å)	Bonding Type	CDOCKER interaction energy (kcal/mol)
3-Butylphthalide 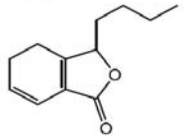	α_1Arg141	2.011	α_1Arg141	1.786	α_2Val1	2.249	H-bond	-29.92
	α_1Arg141	2.048	α_1Arg141	1.955	α_2Val1	2.314	H-bond	
E-Ligustilide 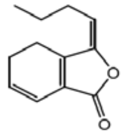	α_1Arg141	2.210	α_2Val1	1.700	α_1Arg141	2.054	H-bond	-24.8
	α_2Val1	1.820			α_1Tyr140	2.324	H-bond	
					α_1Arg141	3.659	π-cation	
Senkyunolide F 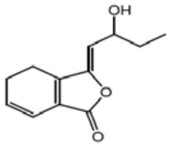	α_1Arg141	1.990	α_1Arg141	2.011	α_1Arg141	2.047	H-bond	-27.55
	α_2Val1	2.184	α_2Val1	1.731	α_1Tyr137	2.417	H-bond	
					α_1Arg141	5.100	π-cation	
					α_2Val1	4.534	π-cation	
Senkyunolide H 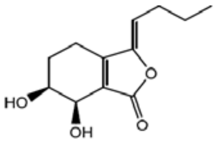	α_1Arg141	1.955	α_1Ser133	2.053	α_1Arg141	2.065	H-bond	-33.21
					α_1Arg141	2.256	H-bond	
	α_1Arg141	2.155			α_2Val1	2.164	H-bond	
	α_1Arg141	2.360			α_2Val1	2.352	H-bond	
	α_2Ser102	1.633			α_1Arg141	3.646	π-cation	
	α_2Ser133	1.754			β_2Trp37	5.779	π-π	
3-Butylidene-4-hydrophthalide 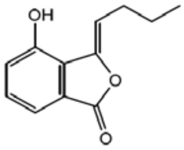	α_1Arg141	1.730	α_1Arg141	1.799	α_1Arg141	2.216	H-bond	-29.36
	α_1Arg141	1.954	α_1Arg141	1.955	α_1Tyr140	2.385	H-bond	
	α_1Thr137	2.048			α_1Arg141	3.446	π-cation	
					α_1Arg141	3.848	π-cation	
					β_2Trp37	5.338	π-π	
6,7-Dihydroxyligustide 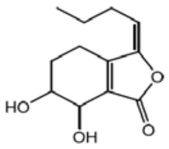	α_2Val1	1.834	α_2Val1	1.800	α_2Val1	1.752	H-bond	-38.56
	α_2Val1	1.964	α_2Val1	1.859	α_2Val1	2.261	H-bond	
	α_1Arg141	2.248	α_1Arg141	2.227	α_2Val1	2.277	H-bond	
	α_2Lys127	2.107	α_2Lys127	2.012	α_1Tyr140	2.374	H-bond	
					α_1Arg141	2.061	H-bond	
					α_1Arg141	2.195	H-bond	
					α_1Ser131	2.294	H-bond	
					α_1Arg141	3.723	H-bond	
					α_2Lys127	6.315	π-cation	

Supplementary References:

1 Ferrari, M. D. Migraine. *Lancet* **351**, 1043-1051, doi:10.1016/s0140-6736(97)11370-8 (1998).

2 Goadsby, P. J., Lipton, R. B. & Ferrari, M. D. Drug therapy: Migraine - current understanding and treatment. *New Engl J Med* **346**, 257-270, doi:10.1056/NEJMra010917 (2002).

3 Altunyurt, S., Gol, M., Sezer, O. & Demir, N. Primary dysmenorrhea and uterine blood flow. *J Reprod Med* **50**, 251-255 (2005).

4 Dawood, M. Y. Primary dysmenorrhea - advances in pathogenesis and management. *Obstet Gynecol* **108**, 428-441, doi:10.1097/01.AOG.0000230214.26638.0c (2006).

5 Marlow, G. Deficient oxygen transport: An alternative mechanism for the development of hypertension? *Am. J Hypertens* **15**, 924-924, doi:10.1016/s0895-7061(02)03024-8 (2002).

6 Hock, C. *et al.* Decrease in parietal cerebral hemoglobin oxygenation during performance of a verbal fluency task in patients with Alzheimer's disease monitored by means of near-infrared spectroscopy (NIRS) - correlation with simultaneous rCBF-PET measurements. *Brain Res.* **755**, 293-303, doi:10.1016/s0006-8993(97)00122-4 (1997).

7 Arai, H. *et al.* A quantitative near-infrared spectroscopy study: A decrease in cerebral hemoglobin oxygenation in Alzheimer's disease and mild cognitive impairment. *Brain Cogn* **61**, 189-194, doi:10.1016/j.bandc.2005.12.012 (2006).

8 Kaminsky, Y. G. *et al.* Age-related defects in erythrocyte 2,3-diphosphoglycerate metabolism in dementia. *Aging Dis* **4**, 244-255, doi:10.14336/AD.2013.0400244 (2013).

9 Elena, A. K., Gjumrakch, A. & Yury, G. K. Relationship between chronic disturbance of 2,3-diphosphoglycerate metabolism in erythrocytes and Alzheimer disease. *CNS Neurol Disord Drug Targets* **15**, 113-123, doi: 10.2174/1871527314666150821103444 (2016).

10 Warburg, O. Origin of cancer cells. *Science* **123**, 309-314, doi:10.1126/science.123.3191.309 (1956).

11 Matsumoto, S. *et al.* Low-field paramagnetic resonance imaging of tumor oxygenation and glycolytic activity in mice. *J Clin Invest* **118**, 1965-1973, doi:10.1172/jci34928 (2008).

12 Chavez, M.D. et al. Structural and functional significance of inhomogeneous line broadening of band III in hemoglobin and Fe-Mn hybrid hemoglobins. *Biochemistry* **29**, 4844-452, doi:10.1021/bi00472a014 (1990).
